# Total and Mitochondrial Transcriptomic and Proteomic Insights into Regulation of Bioenergetic Processes for Shoot Fast-Growth Initiation in Moso Bamboo

**DOI:** 10.3390/cells11071240

**Published:** 2022-04-06

**Authors:** Xiaojing Wang, Xin Geng, Lilin Yang, Yuzhen Chen, Zhiheng Zhao, Weijia Shi, Lan Kang, Ruihua Wu, Cunfu Lu, Jian Gao

**Affiliations:** 1 National Engineering Research Center of Tree Breeding and Ecological Restoration, College of Biological Sciences and Biotechnology, Beijing Forestry University, Beijing 100083, China; wangxj0508@bjfu.edu.cn (X.W.); eyilan@bjfu.edu.cn (X.G.); chenyuzhen@bjfu.edu.cn (Y.C.); zzhhly@bjfu.edu.cn (Z.Z.); shiweijia@bjfu.edu.cn (W.S.); kangLan@bjfu.edu.cn (L.K.); wuruihua@bjfu.edu.cn (R.W.); 2 College of Agriculture and Forestry Engineering and Planning, Tongren University, Tongren 554300, China; ngyyll@gztrc.edu.cn; 3 International Center for Bamboo and Rattan, Key Laboratory of Bamboo and Rattan Science and Technology, State Forestry and Grassland Administration, Beijing 100102, China

**Keywords:** *Phyllostachys edulis*, fast growth, energy metabolism, mitochondrial fission, cold tolerance, proteomics, RNA sequencing

## Abstract

As a fast-growing, woody grass plant, Moso bamboo (*Phyllostachys edulis*) can supply edible shoots, building materials, fibrous raw material, raw materials for crafts and furniture and so on within a relatively short time. Rapid growth of Moso bamboo occurs after the young bamboo shoots are covered with a shell and emerge from the ground. However, the molecular reactions of bioenergetic processes essential for fast growth remain undefined. Herein, total and mitochondrial transcriptomes and proteomes were compared between spring and winter shoots. Numerous key genes and proteins responsible for energy metabolism were significantly upregulated in spring shoots, including those involved in starch and sucrose catabolism, glycolysis, the pentose phosphate pathway, the tricarboxylic acid cycle and oxidative phosphorylation. Accordingly, significant decreases in starch and soluble sugar, higher ATP content and higher rates of respiration and glycolysis were identified in spring shoots. Further, the upregulated genes and proteins related to mitochondrial fission significantly increased the number of mitochondria, indirectly promoting intracellular energy metabolism. Moreover, enhanced alternate-oxidase and uncoupled-protein pathways in winter shoots showed that an efficient energy-dissipating system was important for winter shoots to adapt to the low-temperature environment. Heterologous expression of *PeAOX1b* in Arabidopsis significantly affected seedling growth and enhanced cold-stress tolerance. Overall, this study highlights the power of comparing total and mitochondrial omics and integrating physiochemical data to understand how bamboo initiates fast growth through modulating bioenergetic processes.

## 1. Introduction

Moso bamboo (*Phyllostachys edulis*) is an important forest tree with ecological, cultural and economic importance. As one of the fastest-growing plants on earth, it has attracted much attention worldwide [1,2,3,4,5]. The bamboo’s fast growth is a complicated development process regulated by genetic programs and multiple environmental factors.

Some studies have focused on the fast-growth mechanism of aboveground bamboo culm, observing sequential elongation of internodes from the bottom of the culm to the top and establishing a putative network related to gene regulation, cell behavior, hormone alterations and physiological changes [6,7,8,9,10,11]. In addition, the formation and development of the bamboo rhizome bud is also of great concern [12,13,14,15,16], and this process relies on the parent shoot, rhizome apex and environment [17]. Multiple analyses show that phytohormone signaling, sugar metabolism, meristem development and transcriptional factors coordinate the growth of rhizome buds into shoots [18,19,20].

The shoot stage is the transition phase from initial growth of the rhizome bud to fast growth of the bamboo culm. Similar to seed germination, shoot growth from underground to aboveground is accompanied by great changes, including morphological characteristics and biomass. Nevertheless, the regulation network of this process remains unknown. In general, seed germination is accompanied by an extremely high respiratory rate; a positive correlation between ATP content and germination rate and/or vigor has been found in several plants, supporting the concept that respiratory energy supply is a crucial determinant of germination [21,22]. As well, considering the fact that bamboo shoots are mainly composed of meristematic tissue [23] that conducts rapid cell division [8,24] and consumes much energy, energy metabolism may play a vital role during this transition stage and may be the key to the initiation of fast growth.

Energy for bamboo shoot growth mainly comes from the degradation of sugars such as starch and soluble sugar [25], and mitochondria produce and supply energy through respiration, thus forming a systematic energy-metabolism process [26]. However, details of this process remain unclear, including mitochondrial respiration characteristics, the mitochondrial gene expression profile, and the coordinated expression of mitochondrial genes and nuclear genes. Therefore, it is necessary to extract mitochondria from tissues and use omics to study the mitochondrial energy-metabolism mechanism. At present, this method is mostly used in humans [27], animals and fungi [28] but rarely in plants [29,30,31,32]. Mitochondrial multi-omics analysis will be helpful to identify respiratory pathways, discover key energy genes, and uncover unique mitochondrial energy metabolism regulatory networks during shoot growth, which will fill the gap in bamboo research in this field.

Shoot buds of Moso bamboo, the rudiments of the whole bamboo lifecycle, usually form in summer (June–August) and grow slowly underground for up to 9–10 months to develop into winter shoots. By the following spring (early April), the young winter shoots start to develop rapidly into spring shoots with large size and emerge out of the ground, at which point they grow rapidly into fully developed culms within a short time [25]. In this study, we successfully isolated mitochondria from winter shoots and spring shoots of Moso bamboo and then performed comparative total and mitochondrial transcriptomic and proteomic analyses. The physiological indexes of bamboo shoots related to energy metabolism were also characterized in terms of their contents of starch, soluble sugars and ATP, number of mitochondria, mitochondrial respiration rate and glycolysis rate. The *PeAOX1b* gene underwent heterologous transformation into Arabidopsis for energy dissipation and cold-resistance analysis. The dataset obtained in the current work provides a basis for the energy regulation mechanism of rapid-growth initiation in Moso bamboo.

## 2. Materials and Methods

### 2.1. Sampling of Moso Bamboo Shoots

Moso bamboo materials were planted in the natural forest located in Longsheng county, Guangxi province, China (110°14′18.28″ E, 25°49′50.07″ N; 712 m elevation), where mean annual precipitation is 1544 mm and annual air temperature is 18.1 °C. Moso bamboo shoots were harvested at 9–10 am during the growing season (24 December 2018 for winter shoots and 4 March 2019 for spring shoots) (Figure S1) and transported to the laboratory on ice within 5 h post-harvest. Bamboo shoots were selected based on uniformity of size, color and shape, and absence of any disease or blemish. The winter shoot is about 20 cm long and grows underground, and the spring shoot is about 25 cm long and grows just out of the surface of the ground. For both developmental stages, three samples from different rhizomes were collected as three biological replicates. Considering the asymmetry and unevenness of bamboo shoots from top to bottom, the middle part of the bamboo shoots was used for physiological indexes, RT-qPCR, RNA sequencing, proteome analyses and PRM verification. Part of the fresh tissue was immediately used for measurement of physiological indexes, and the remainder was stored at −80 °C for further use.

### 2.2. Measurement of Physiological Indexes

The contents of starch, soluble sugars and ATP in Moso bamboo shoots were determined using the relevant kits (Naijing Jiancheng Bioengineering Institute, Nanjing, China). The measurements of mitochondrial respiration rate and glycolysis rate were conducted using an XF96 extracellular flux analyzer (Seahorse Bioscience, Billerica, MA, USA) according to a previously described protocol [33] with some modifications (see Supplementary Method S1).

### 2.3. Mitochondrial Isolation and Detection of Mitochondrial Activity, Purity and Count

Mitochondria were isolated from winter shoots and spring shoots (see Supplementary Method S2). Mitochondria-specific dyes were used to identify mitochondrial activity (see Supplementary Method S3), and the active mitochondria showed a distinct blue–green color after staining with Janus green B (Figure S2a,b) and emitted a distinct yellow–green fluorescence after staining with Rhodamine 123 (Figure S2c,d). Moso bamboo nuclear gene *actin* (1131 bp), mitochondrial gene *atp8* (468 bp) and chloroplast gene *psbB* (1527 bp) were used as markers to confirmed the separation of mitochondria from nuclear and chloroplast contaminants by PCR amplification (Figure S2e,f). The specific primers of marker genes are listed in Table S1. Mitochondrial count was determined according to previously described protocol [34,35] with some modifications (see Supplementary Method S4).

### 2.4. RNA-Seq Analysis

Total RNA was isolated separately from winter shoots, spring shoots, winter shoot mitochondria and spring shoot mitochondria using a TaKaRa MiniBEST plant RNA extraction kit (TaKaRa Bio, Nojihigashi, Japan) to construct a total of 12 cDNA libraries. The quantified cDNA libraries were then sequenced on the Illumina HiSeq X ten platform (Illumina, San Diego, CA, USA). After removing the adaptor sequences and low-quality sequences from the raw reads, the remaining clean reads from winter and spring shoot samples were mapped to the Moso bamboo reference genome [1], and the clean reads from mitochondria of winter and spring shoot samples were mapped to the mitochondrial reference genome of bamboo *Bambusa oldhamii* (accession number: EU365401). Only uniquely mapped reads were retained for subsequent analysis. The normalized expression levels for gene models were measured as FPKM (fragments per kilobase of transcript per million mapped reads) [36]. Differentially expressed genes (DEGs) were identified with the following default criteria: fold change > 2 and *p*-value < 0.05. TIGR Multiple Experiment Viewer (MeV 4.9.0) software was used for hierarchical clustering. A *p*-value ≤ 0.05 was used as the threshold to define significantly enriched Gene Ontology (GO) term or Kyoto Encyclopedia of Genes and Genomes (KEGG) pathways associated with the DEGs [37].

### 2.5. RT-qPCR Analysis

Total RNA was isolated from bamboo shoot samples and mitochondrial samples, and RT-qPCR was performed as previously described [38]. Both Moso bamboo *actin* and *TIP41* genes were used as internal controls [3,18] for bamboo shoot samples, and both Moso bamboo *rps13* and *ccmFC* genes were used as internal controls for mitochondrial samples. PCR-specific primers of target genes are listed in Table S2, and the 2^−*ΔΔ*Ct^ comparative-threshold-cycle (Ct) method [39] was applied to calculate the relative expression levels of target genes.

### 2.6. Label-Free Quantitative Proteomics and PRM Verification

The twelve samples from the same source as the transcriptomics were used for label-free quantitative proteomics. Protein extraction and digestion, LC-MS analysis, protein identification and relative quantification were carried out as described previously [40]. Protein annotation was performed against the transcriptome database we obtained in this study. Correlation values between samples were calculated according to the quantitative results. Differentially expressed proteins (DEPs) were identified as having significance thresholds of fold change ≥ 2 and *p* < 0.05. GO and pathway enrichment analyses as well as clustering analysis for DEPs were performed as previously described for DEGs.

The targeted proteins were selected for validation by parallel-reaction monitoring (PRM) on a Q Exactive HFX mass spectrometer (Thermo, Waltham, MA, USA) coupled with the U3000 system (DIONEX, Sunnyvale, CA, USA), with further details in Supplementary Method S5.

### 2.7. Gene Cloning, Plasmid Construction, Transformation and Screening for Homozygous Lines

Based on the *PeAOX1b* sequence, we designed the forward primer (5′-GGTACCATGCCCGCGGCCGCGAGGAT-3′, containing site for restriction enzyme KpnI) and reverse primer (5′-TCTAGAGTGATACCCGAGCGGCGCGG-3′, containing site for restriction enzyme XbaI) and amplified the full-length coding sequence of *PeAOX1b* by PCR, utilizing cDNA from Moso bamboo leaf as the template. The PCR product was digested with KpnI/XbaI, then cloned into a pCAMBIA2300-GFP reconstructed vector that uses the CaMV35S promoter to drive expression. Recombinant plasmid was transferred into Agrobacterium tumefaciens strain GV3101 and used for transforming Arabidopsis via the floral dip method. T1–T3 transgenic Arabidopsis seeds were germinated on sterile medium containing kanamycin (50 μg mL^−1^) to screen positive plants, RT-PCR was performed to identify T3 homozygous lines (Figure S3a). Image J software was used to further analyze the gray values of electrophoretic bands of RT-PCR in Figure S3a, and the relative expression levels of the *PeAOX1b* gene in transgenic lines were counted based on the ratio of gray values (Figure S3b). Finally, T3 generation seeds and seedlings were used for cold-stress tolerance analysis.

### 2.8. Cold-Stress Tolerance Analysis

The wild-type and transgenic Arabidopsis seeds were first vernalized at 4 °C on MS medium (pH 5.8) for three days and then cultured in a growth chamber at 22 °C under a 16 h photoperiod and 150 μmol m^−2^ s^−1^ light intensity. For cold-tolerance evaluation, 17-day-old wild-type and transgenic Arabidopsis seedlings cultured under normal conditions were transferred to 4 °C for three days. Leaf respiration test was performed on single tissue disc with 2.5-mm-diameter in leaf center using the same method as for Moso bamboo shoots. The MDA content, Pro content and SOD activity in leaves were measured using the relevant kits (Naijing Jiancheng Bioengineering Institute, Nanjing, China), and the electrolyte leakage rate (ELR) of leaves was tested according to the previous method [41] with some modifications (see Supplementary Method S6). Each experiment was re

### 2.9. Statistical Analysis

Experimental data were presented as mean ± standard deviation (SD) of three independent replicates, and the GraphPad Prism 8 software (GraphPad v8.0.2 https://www.graphpad.com/guides/prism/8/user-guide/index.htm?whats-new-highlights.htm (accessed on 21 December 2018)) was used to plot graphs. Based on IBM SPSS Statistics 21 software (IBM v21.0 https://www.ibm.com/support/pages/spss-statistics-210-available-download (16 April 2020)), the data were analyzed for normality and homogeneity; when they met the assumptions, the Duncan method of single-factor ANOVA was used to compare the significant level of difference (*p* < 0.05 or *p* < 0.01).

## 3. Results

### 3.1. Enhanced Repiration and Energy Production and Mitochondria Biogenesis in Spring Shoots

**Figure 1:**
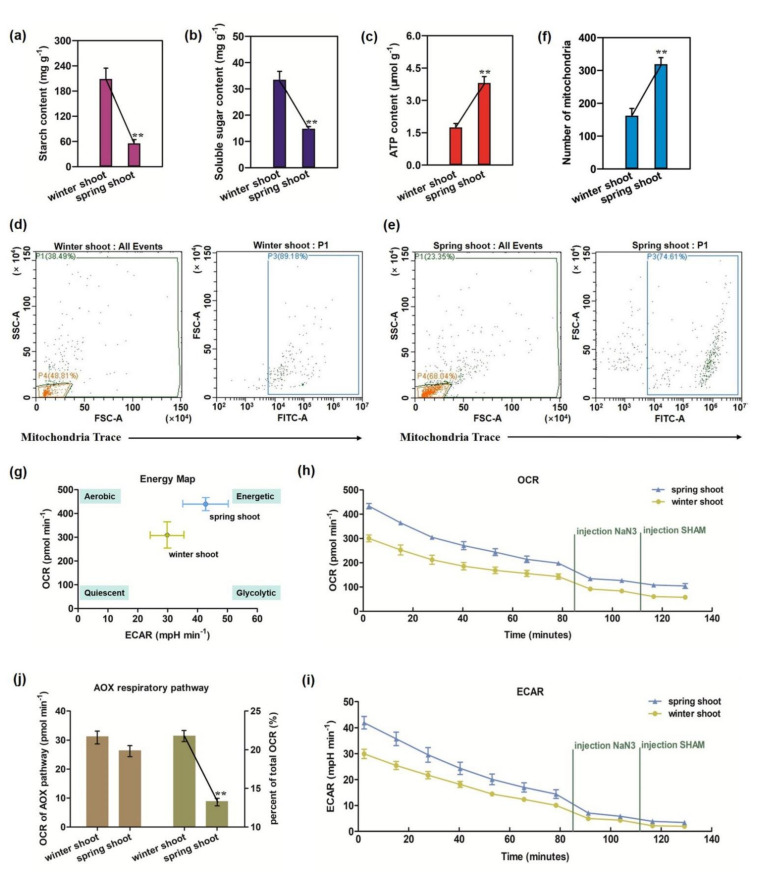
Analysis of physiological indexes related to energy metabolism in different development stages of Moso bamboo shoots. (**a**) Starch content analysis. (**b**) Soluble sugar content analysis. (**c**) ATP content analysis. (**d**,**e**) Mitochondria were sorted by CytoFLEX of 100 μL purified mitochondria suspensions of winter and spring shoots (P1, all particles; P3, mitochondrial particles; P4, noise data). (**f**) Number of mitochondrial particles per 100 μL of purified mitochondrial suspension. (**g**) The initial OCR and ECAR of winter and spring shoots. (**h**,**i**) Changes to OCR and ECAR after adding COX and AOX respiratory inhibitors NaN3 and salicylhydroxamic acid (SHAM) into the respiration buffer. (**j**) OCR of AOX respiratory pathway and its proportion in total OCR. ** represents significant difference *p* < 0.01.

As shown in Figure 1a,b, starch and soluble sugar contents in spring shoots decreased significantly (*p* < 0.01)—by 73% and 56%, respectively—while ATP content significantly increased by 117% (Figure 1c), indicating that the catabolism of starch and soluble sugar was increased and more ATP was produced in spring shoots. The number of mitochondria in winter and spring shoots was counted by CytoFLEX (Beckman, Brea, CA, USA) (Figure 1d,e), and spring shoots presented a mean concentration of approximately 319 mitochondrial particles per 100 μL of purified mitochondrial suspension (Figure 1f), nearly twice that of winter shoots.

Mitochondrial respiration and glycolysis are two important ATP-production pathways, and their metabolic intensity can be characterized by oxygen-consumption rate (OCR) and extracellular-acidification rate (ECAR), respectively. We found that the initial OCR and ECAR values (432.93 pmol min^−^^1^ and 41.93 mpH min^−^^1^, respectively) of spring shoots were much higher than that of winter shoots (300.22 pmol min^−1^ and 29.94 mpH min^−^^1^, respectively) (Figure 1g), indicating that mitochondrial respiration and glycolysis were obviously enhanced in spring shoots.

Different respiratory pathways of mitochondria were identified using various respiratory inhibitors. The OCR and ECAR of Moso bamboo shoots were inhibited simultaneously by the injection of the cytochrome c oxidase (COX) respiratory poison NaN3 into the respiration buffer (Figure 1h,i). The addition of the alternative oxidase (AOX) respiratory pathway inhibitor salicylhydroxamic acid (SHAM) further inhibited OCR and ECAR. The changes to OCR before and after the addition of different inhibitors (Table S3) showed that the OCR value of AOX pathway in winter shoots (31.21 pmol min^−1^) was higher than that of spring shoots (26.37 pmol min^−1^) (Figure 1j). Moreover, the proportion of AOX respiratory pathway to total OCR in winter shoots (21.79%) was significantly (*p* < 0.01) higher than that in spring shoots (13.31%). Taken together, the respiratory activity of the AOX pathway was stronger in winter shoots than that in spring shoots.

### 3.2. The Systemic Energy Generation Pathway Was Activated at the Transcriptional Level during Shoot Growth

**Figure 2:**
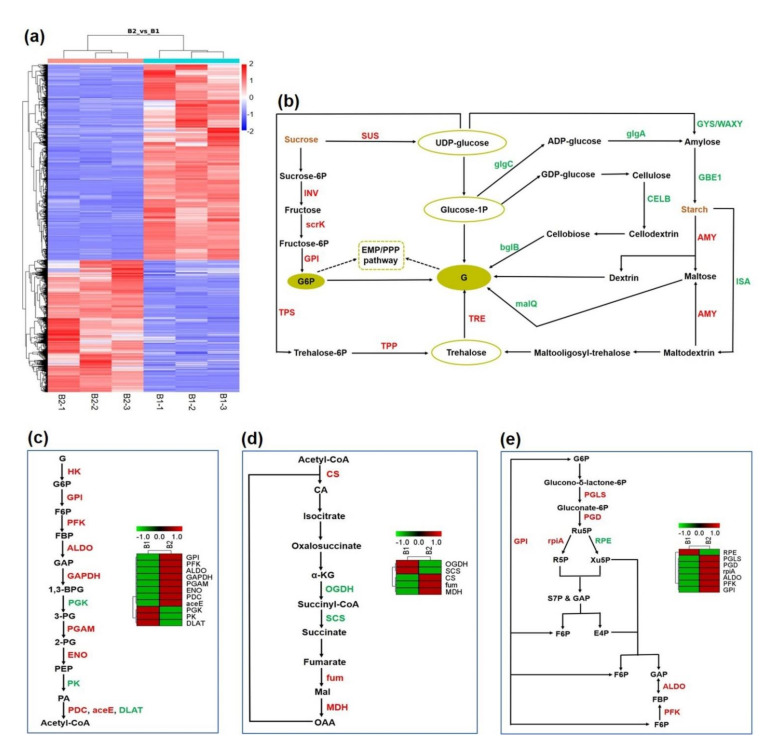
Differential expression analysis in total transcriptome. (**a**) Cluster analysis of differentially expressed genes. (**b**–**e**) Expression changes of key enzyme genes involved in different energy metabolism pathways at two development stages. Key players in starch and sucrose metabolism (**b**), EMP (**c**), TCA cycle (**d**) and PPP (**e**) pathway: the red letters represent upregulated genes and the green letters indicate downregulated genes. Abbreviations: B1, winter shoots; B2, spring shoots; G, glucose; G6P, glucose−6−phosphate; SUS, sucrose synthase; INV, invertase; GPI, glucose−6−phosphate isomerase; scrK, fructokinase; TPP, trehalose 6−phosphate phosphatase; TRE, alpha, alpha-trehalase; TPS, trehalose 6−phosphate synthase; GYS, glycogen synthase; WAXY, granule-bound starch synthase; GBE1, 1,4−alpha-glucan branching enzyme; AMY, amylase; ISA, isoamylase; malQ, 4−alpha-glucanotransferase; glgA, starch synthase; glgC, glucose−1−phosphate adenylyltransferase; CELB, cellulase; bglB, beta-glucosidase; HK, hexokinase; F6P, fructose−6−phosphate; GPI, glucose-6−phosphate isomerase; PFK, phosphofructkinase; FBP, fructose−1,6−bisphosphatase; ALDO, fructose-bisphosphate aldolase; GAP, fructose-bisphosphate aldolase; GAPDH, glyceraldehyde phosphate dehydrogenase; 1, 3−BPG, 1, 3−bisphosphoglycerate; PGK, phosphoglycerate kinase; 3−PG, 3−phosphoglycerate; PGAM, phosphoglycerate mutase; 2−PG, 2−phosphoglycerate; ENO, enolase; PEP, phosphoenolpyruvate; PK, pyruvate kinase; PDC, pyruvate decarboxylase; aceE, pyruvate dehydrogenase E1 component; DLAT, pyruvate dehydrogenase E2 component; CS, citrate synthase; CA, citric acid; α-KG, α-ketoglutarate; SCS, succinyl-CoA synthetase; OGDH, oxoglutarate dehydrogenase; Mal, malate; fum, fumarase; MDH, malate dehydrogenase; OAA, oxaloacetic acid; PGLS, 6−phosphogluconolactonase; PGD, 6−phosphogluconate dehydrogenase; rpiA, ribose 5−phosphate isomerase A; Ru5P, ribulose 5−phosphate; RPE, ribulose-phosphate 3−epimerase; Xu5P, xylulose 5−phosphate; R5P, ribose 5−phosphate; S7P, sedoheptulose 7−phosphate; E4P, erythrose 4−phosphate. The color scale represents the Z-score calculated.

Total transcriptome analysis was performed on samples of winter shoots (B1) and spring shoots (B2). An average of 83.10% of clean reads from each library mapped uniquely to the reference genome (Table S4). A total of 4897 upregulated DEGs and 7261 downregulated DEGs were identified by taking B1 as the control (Figure S4), and DEG cluster analysis showed good repeatability among the samples (Figure 2a). GO and KEGG enrichment analyses were conducted to understand the major functional changes during shoot growth. Remarkably, the most significantly enriched functions involved several mitochondrial and sugar-metabolism functions: mitochondrial RNA modification, mitochondrial RNA metabolic processes, sucrose synthase activity (Figure S5) and starch and sucrose metabolism (Figure S6). These results support the concept that shoot growth causes universal changes in gene expression associated with energy production.

As the energy source of shoot growth, starch and sucrose are produced by above-ground tissues through photosynthesis and transported to underground tissues [25]. In the total transcriptome, many important enzyme genes responsible for starch and sucrose catabolism were significantly upregulated (Figure 2b, Table S5), such as sucrose synthase (*SUS*), invertase (*INV*) and amylase (*AMY*). On the other hand, enzyme genes directed toward starch and sucrose synthesis were significantly downregulated (Table S5), such as starch synthase (*glgA*), glycogen synthase (*GYS*), granule-bound starch synthase (*WAXY*) and 1,4-alpha-glucan branching enzyme (*GBE1*), which may lead to a large decrease in starch and sucrose content and produce more glucose (G) and glucose-6-phosphate (G6P) for further oxidation in the carbohydrate metabolism pathway.

Next, three major carbohydrate metabolism pathways were compared and analyzed: glycolysis (EMP) (Figure 2c), the tricarboxylic acid (TCA) cycle (Figure 2d) and the pentose phosphate pathway (PPP) (Figure 2e). In this study, a total of 21 kinds of enzyme genes were identified in these three pathways, 15 of which were upregulated. The significantly upregulated expression of these enzymes, such as hexokinase (*HK*, 27-fold), phosphofructkinase (*PFK*, 6-fold), glyceraldehyde phosphate dehydrogenase (*GAPDH*, 7-fold) in EMP; citrate synthase (*CS*, 2-fold), fumarase (*fum*, 2-fold) and malate dehydrogenase (*MDH*, 2-fold) in the TCA cycle; and 6-phosphogluconolactonase (*PGLS*, 4-fold), 6-phosphogluconate dehydrogenase (*PGD*, 2-fold) and ribose 5-phosphate isomerase A (*rpiA*, 4-fold) in PPP; showed positive correlation with ATP content. Taken together, these data suggest that EMP, the TCA cycle and the PPP were activated at the spring-shoot stage, providing sufficient energy for fast-growth initiation.

Furthermore, eleven DEGs related to energy metabolism were selected for RT-qPCR analysis (Figure S7). As expected, RT-qPCR results confirmed the expression pattern observed in DEG analysis by RNA-seq, indicating that total transcriptome data was highly reliable in the present study.

### 3.3. The Unique Gene-Expression Profile and Energy-Metabolism System in Mitochondria

**Figure 3:**
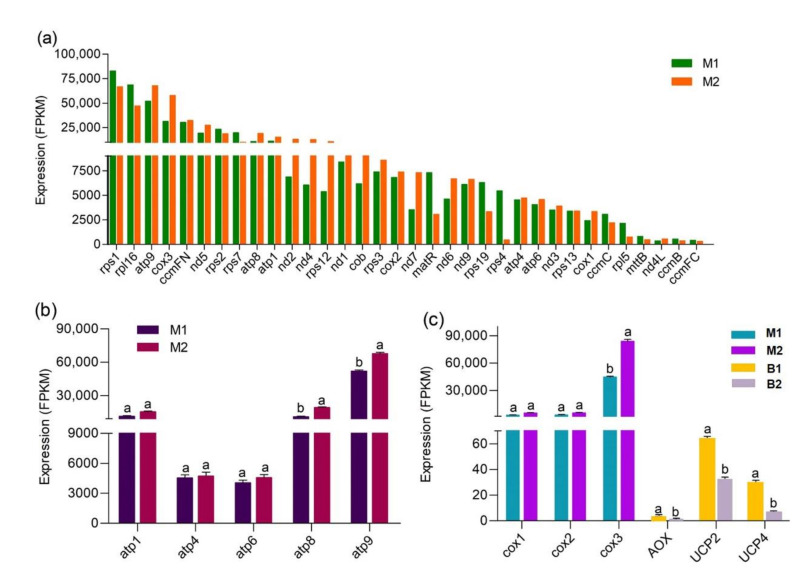
Differential expression analysis. (**a**) Quantification level of mitochondrial-protein-coding genes. (**b**) Expression level of mitochondrial ATP synthase genes at different stages. (**c**) Comparative analysis of expression level of key genes in different mitochondrial energy-metabolism pathways. Abbreviations: M1, winter shoot mitochondria; M2, spring shoot mitochondria; B1, winter shoots; B2, spring shoots. The letters a and b in the graph indicate significant differences (*p* < 0.05).

Mitochondrial transcriptome analysis was performed on samples from winter shoot mitochondria (M1) and spring shoot mitochondria (M2). An average 2.56% of clean reads from each library mapped uniquely to the mitochondrial genome (Table S6). In total, 34 protein-coding genes were identified, showing nearly the same coding capability as mitochondrial genomes of other grasses (Table S7). Besides, 22 genes were more highly expressed in M2 than in M1 (Figure 3a); these genes were closely related to electron transport and ATP synthesis.

To obtain the co-expressed mitochondrial genes in the total and mitochondrial transcriptome, we screened the mitochondrial genes in the sequencing results of B1 and B2, finally identifying only eight genes encoded by the mitochondrial genome (Figure S8). These eight genes showed the same expression trends but varied sharply in expression level between M1_M2 and B1_B2, which reflects the obvious effect of purification and enrichment of mitochondria from bamboo shoots.

KEGG-enrichment analysis revealed that mitochondrial DEGs were involved in two energy metabolic pathways: oxidative phosphorylation (OXPHOS) and thermogenesis. The standard mitochondrial electron transfer chain in plants consists of four multi-protein complexes, known as complexes I, II, III, and IV [42]. When the substrates NADH and succinate are oxidized, electrons are transferred from complexes I and II to complex III via coenzyme-Q (CoQ), from complex III to complex IV via cytochrome c, and finally, to O2. During this process, the protons (H^+^) in the mitochondrial matrix are pumped to the intermembrane space, resulting in a proton electrochemical potential coupled to the conversion of adenosine diphosphate (ADP) and Pi to ATP, catalyzed by complex V [43]. In the current transcriptome, no complex II subunits (*sdh3* and *sdh4*) were detected, this is consistent with the loss of the two genes in the mitochondrial genomes of other grasses (Table S7), suggesting that CoQ can only obtain electrons from complex I. Further, all enzyme genes involved in OXPHOS, especially ATP synthase genes (Figure 3b), were upregulated at the spring-shoot stage, and eleven genes assayed by RT-qPCR closely matched mitochondrial RNA-seq results (Figure S9). These and related observations suggested that OXPHOS was activated in spring shoots, resulting in a large amount of ATP production to initiate rapid growth of Moso bamboo.

In addition to the standard pathway, AOX can transfer electrons from CoQ to O2 and operate a non-phosphorylating bypass mechanism [44], and UCP (uncoupling protein) can uncouple the process by which H^+^ returns to the mitochondrial matrix from the intermembrane space with ATP synthesis [45], both of which dissipate free energy as heat and are named thermogenesis. The *AOX* and *UCP* genes were identified in the total transcriptome, but their expression levels were much lower than *cox* (Figure 3c), indicating these two pathways are not dominant. RNA-seq and RT-qPCR results indicated that the expression levels of *AOX* and *UCP* genes were significantly higher in winter shoots (Figure S7), implying more heat was produced in winter-shoot mitochondria.

### 3.4. Enhanced Expression of Genes Related to Mitochondrial Fission in Spring Shoots

**Figure 4:**
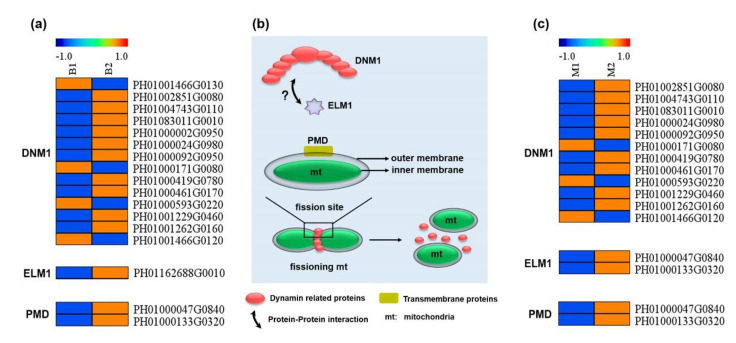
DEGs related to mitochondrial fission and the model of mitochondrial fission during shoot growth in Moso bamboo. Key DEGs related to mitochondrial fission in total (**a**) and mitochondrial (**c**) transcriptome. (**b**) The model of mitochondrial fission during shoot growth in Moso bamboo.

Mitochondrial fission depends on the regulation of the dynamin gene [46]. Fourteen putative dynamin 1 (*DNM1*) genes in the total transcriptome were identified, and ten of them were upregulated in spring shoots (Figure 4a). Further, peroxisomal and mitochondrial division factor (*PMD*), elongated mitochondria1 (*ELM1*) and two plant-specific fission factors were found to be upregulated in spring shoots. In addition, similar results were found in the mitochondrial transcriptome (Figure 4c), suggesting that the mitochondrial fission activity in spring shoots was enhanced.

### 3.5. The Systemic Energy Generation Pathway Was Induced at the Protein Level during Shoot Growth

Label-free quantitative proteomics were performed to compare the differences between total and mitochondrial proteome during shoot growth. Collectively, 6141 proteins were identified, of which 3381 were common in B1, B2, M1 and M2, corresponding to 226, 76, 129 and 138 unique proteins, respectively (Figure S10a). Based on their quantitative values, the global expression profile of the proteins was characterized via cluster heatmap (Figure S10b), and the correlation value (≥ 0.82) between samples revealed a good biological repeatability (Figure S10c). Remarkably, a greater number of upregulated differentially expressed proteins (DEPs) were screened out in the mitochondrial proteome (934) than in the total proteome (441) (Figure S10d,e).

Similar to transcriptome analysis, multiple significantly enriched GO and KEGG functions at the protein level were also involved in mitochondrial and sugar metabolism functions, including mitochondrial part, mitochondrial matrix, starch and sucrose metabolism and carbohydrate catabolic process in the total proteome (Figures S11 and S12), and NADH dehydrogenase activity and energy derivation by oxidation of organic compounds in the mitochondrial proteome (Figures S13 and S14). These results indicate that protein expression changes related to energy generation generally occurred during shoot growth.

Compared to the total transcriptome, fewer types and quantities of enzyme proteins responsible for starch and sucrose metabolism were identified from the total proteome (Table S8), including WAXY, glgA, trehalose 6-phosphate synthase (TPS), beta-glucosidase (bglB), glucose-1-phosphate adenylyltransferase (glgC) and 4-alpha-glucanotransferase (malQ). The significantly downregulated expression of WAXY, glgA and glgC proteins would lead to a reduction in starch synthesis, while the significantly upregulated expression of bglB protein was conducive to the formation of glucose.

In total, 15 DEPs responsible for carbohydrate and mitochondrial energy metabolism were identified from the total proteome (Table 1). Regarding the abundance change of these proteins, 40S ribosomal protein S3a (rpiA), phosphoglucomutase (pgm), pyruvate kinase (PK) and dehydrogenase E1 component domain containing protein (aceE) showed decreased expression, while the remaining 11 proteins showed significantly increased expression greater than 2-fold. In addition, except for 2Fe-2S iron-sulfur cluster binding domain containing protein (NDUFS1) and 4Fe-4S binding domain containing protein (NDUFS8), the expression trends of most proteins in different developmental stages of Moso bamboo shoots were consistent with the transcription level.

### 3.6. More Proteins Related to Energy Metabolism and Mitochondrial Fission Were Identified in the Mitochondrial Proteome

Proteins extracted from the mitochondria-enriched fractions displayed a total of 23 DEPs related to carbohydrate and mitochondrial energy metabolism (Table 2), far more than that of the total proteome. Only 4 proteins showed downregulated expression, while the remaining 19 proteins showed different degrees of significantly upregulated expression, especially H^+^-transporting ATPase (PMA, 5-fold), F-type H^+^-transporting ATPase subunit epsilon (ATPF1E, 823-fold) and F-type H^+^-transporting ATPase subunit beta (ATPeF1B, 33-fold). Further, a DNM1 protein involved in mitochondrial fission was identified to be significantly upregulated.

To confirm the protein expression levels obtained by Laber-free, 20 of 23 candidate proteins related to carbohydrate and mitochondrial energy metabolism in the mitochondrial proteome were selected for PRM analysis. On the whole, the protein expression trends determined by PRM and label-free were largely consistent (Table 2), indicating that our label-free results were reliable and reproducible.

### 3.7. Conjoint Analysis Revealed the Energy Metabolic Mechanism of Fast-Growth Initiation in Bamboo Shoots

**Figure 5:**
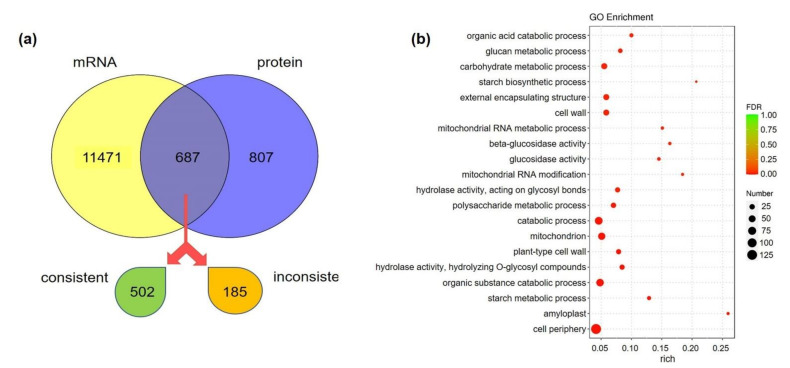
Conjoint analyses of transcriptome and proteome. (**a**) Correlation analysis of numbers of DEGs and DEPs. (**b**) GO functional-enrichment analysis of the differentially expressed correlations (DECs): *X* axis indicates rich factor; *Y* axis means GO terms. Rich factor is the ratio of DEC number, annotated in this term to all gene numbers. Greater rich factor represents greater intensiveness. The number of DECs is represented by the size of the circle, with a larger circle indicative of more DECs. FDR is false discovery rate ranging from 0–1, with a lower FDR indicative of greater intensiveness. Only the top 20 enriched terms are displayed.

To gain insight into the relationship between transcription and translation during shoot growth, transcriptome and proteome data were combined for further analysis. The number of DEGs and DEPs differed widely, with only 687 differentially expressed correlations (DECs) (Figure 5a), 502 of which showed consistent trends in expression at both transcription and protein levels. Among the top 20 DECs with the most significant differences, 16 trended similarly in both transcription level and protein abundance (Figure S15). Moreover, the significant enrichment functions of DECs were largely related to mitochondria and sugar metabolism (Figure 5b and Figure S16), which was consistent with the transcriptome and proteome analyses.

**Figure 6:**
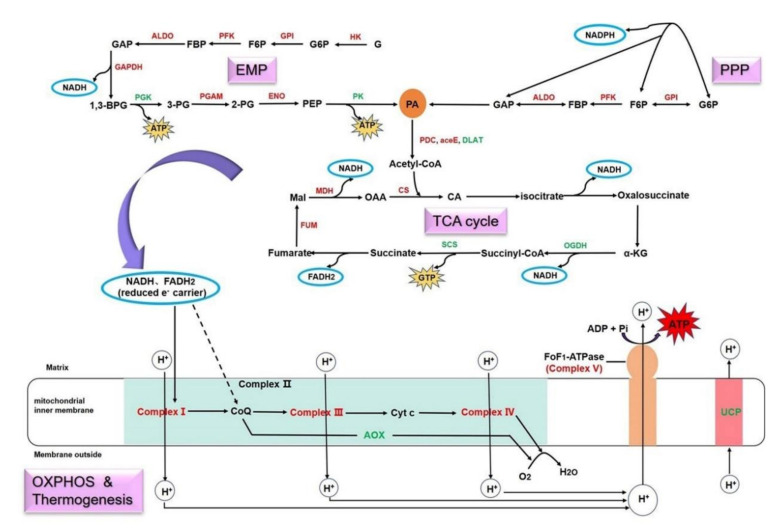
Energy metabolic mechanism for fast-growth initiation in Moso bamboo shoots. The red letters represent upregulated genes and the green letters indicate downregulated genes. Abbreviations: EMP, glycolysis; PPP, pentose phosphate pathway; OXPHOS, oxidative phosphorylation; TCA, tricarboxylic acid; NADH, reduced nicotinamide adenine dinucleotide; FADH2, reduced flavin adenine dinucleotide; ATP, adenosine triphosphate; NADPH, reduced nicotinamide adenine dinucleotide phosphate; GTP, guanosine triphosphate; ADP, adenosine diphosphate; Pi, phosphate group; G, glucose; HK, hexokinase; GPI, glucose−6−phosphate isomerase; PFK, phosphofructkinase; ALDO, fructose-bisphosphate aldolase; PGK, phosphoglycerate kinase; GAPDH, glyceraldehyde phosphate dehydrogenase; ENO, enolase; PGAM, phosphoglycerate mutase; PK, pyruvate kinase; G6P, glucose−6−phosphate; F6P, fructose−6−phosphate; FBP, fructose−1,6−bisphosphatase; GAP, fructose-bisphosphate aldolase; 1,3−BPG, 1,3−bisphosphoglycerate; 2−PG, 2−phosphoglycerate; 3−PG, 3−phosphoglycerate; PEP, phosphoenolpyruvate; PA, pyruvic acid; PDC, pyruvate decarboxylase; aceE, pyruvate dehydrogenase E1 component; DLAT, pyruvate dehydrogenase E2 component; CS, citrate synthase; CA, citric acid; α-KG, α-ketoglutarate; SCS, succinyl-CoA synthetase; OGDH, oxoglutarate dehydrogenase; fum, fumarase; Mal, malate; MDH, malate dehydrogenase; OAA, oxaloacetic acid; Complex I, NADH dehydrogenase; Complex II, succinate dehydrogenase; Complex III, cytochrome reductase; Complex IV, cytochrome oxidase; Complex V, ATP synthase; CoQ, coenzyme-Q; Cyt c, cytochrome c; AOX, alternate oxidase; UCP, uncoupled protein; H^+^, proton; O2, oxygen; H2O, water.

The energy needed for bamboo-shoot growth mainly comes from carbohydrate oxidation, which consists of four main pathways: EMP, PPP, TCA cycle and OXPHOS (Figure 6). EMP and PPP occur in the cytoplasm; their main function is to break down glucose into PA and produce NADH, reduced nicotinamide adenine dinucleotide phosphate (NADPH) and a tiny amount of ATP. The TCA cycle, which occurs in the mitochondria, is a further oxidation process of PA, producing guanosine triphosphate (GTP), NADH and reduced flavin adenine dinucleotide (FADH2). As the carriers of electrons, these NADH and FADH2 from different pathways enter the electron transport chain in the mitochondrial inner membrane and initiate OXPHOS and ATP production. In this study, most of the DEGs and DEPs involved in these pathways (especially OXPHOS) were upregulated, indicating that carbohydrate oxidation was enhanced at the spring-shoot stage. Of concern was that the absence of complex II caused NADH to become the primary source of electrons. In addition to the standard respiratory pathway, the AOX pathway and UCP pathway were also identified during shoot growth. In spite of inapparent pathways, they might permit routes for heat production in response to cold stress, especially during the winter-shoot stage.

### 3.8. Heterologous Expression of PeAOX1b in Arabidopsis Affected Seedling Growth and Enhanced Cold-Stress Tolerance

**Figure 7:**
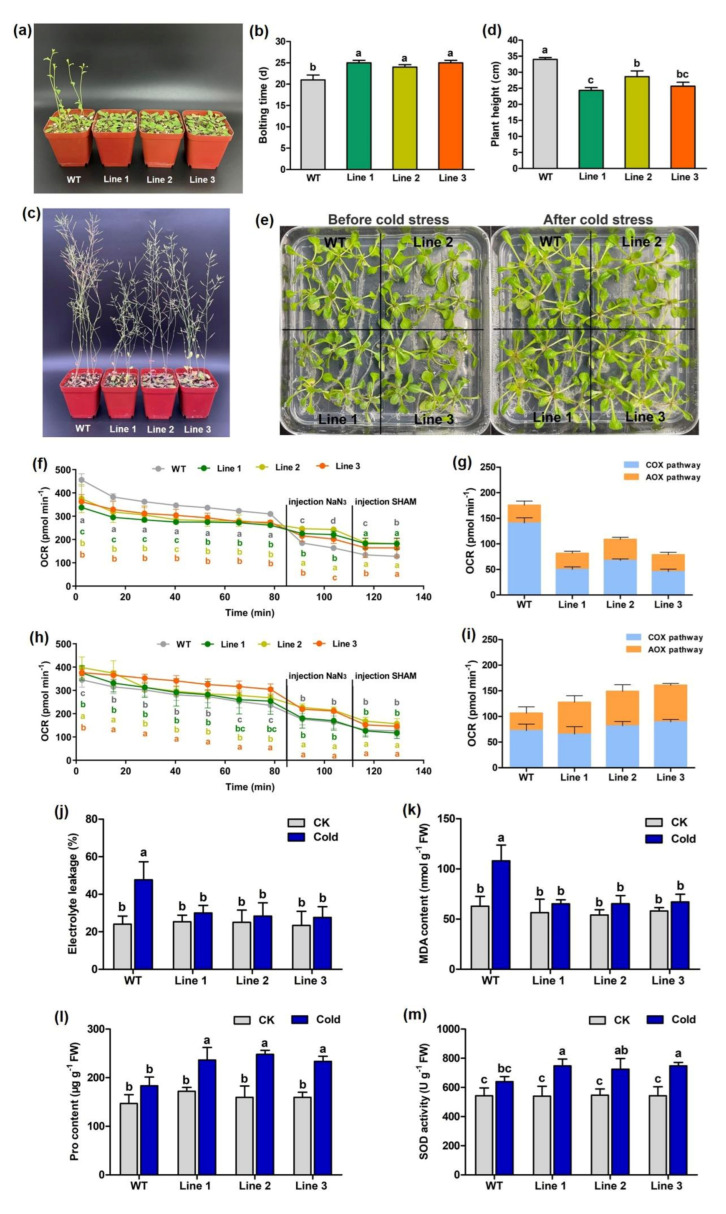
Phenotypes and cold resistance analysis of WT and *PeAOX1b* transgenic Arabidopsis. (**a**) WT and transgenic Arabidopsis cultured for 23 days. (**b**) Bolting time of WT and transgenic Arabidopsis. (**c**) WT and transgenic Arabidopsis cultured for 40 days. (**d**) Plant height of WT and transgenic Arabidopsis cultured for 40 days. (**e**) WT and transgenic Arabidopsis seedings before and after cold stress. (**f**) Determination of OCR and different respiratory pathways under normal conditions. The letters a, b, c, d in the graph indicate significant differences between different Arabidopsis lines. (**g**) The proportion of different respiratory pathways in leaves of different Arabidopsis lines. (**h**) Determination of OCR and different respiratory pathways under cold stress. The letters a, b, c, d in the graph indicate significant differences between different Arabidopsis lines. (**i**) The proportion of different respiratory pathways in leaves of different Arabidopsis lines under cold stress. The electrolyte leakage rate (**j**), MDA content (**k**), Pro content (**l**) and SOD activity (**m**) in leaves of WT and transgenic Arabidopsis under cold treatment. The average and standard deviation (SD) come from three biological replicates. Significant difference criteria: *p* < 0.05. Abbreviations: WT, wild type Arabidopsis; Line1−Line3, transgenic Arabidopsis of different lines; OCR, oxygen consumption rate.

Swiss-Prot and Pfam database annotations indicated that the *AOX* gene detected from our transcriptome belonged to member *AOX1b* of the *AOX* gene family. To characterize *PeAOX1b*, transgene experiments were performed. Phenotypic analysis showed that the bolting times of transgenic seedlings were significantly (*p* < 0.05) later than that of the wild-type (WT) (Figure 7a,b), and the plant height of transgenic seedlings were significantly shorter than the WT ones after 40 days of growth (Figure 7c,d). Compared to WT plants, total OCR decreased significantly in transgenic lines (Figure 7f), but the proportion of the AOX pathway in total OCR increased significantly in transgenic seedlings (Figure 7g). These results implied that *PeAOX1b* plays an important role in the respiratory system, mainly promoting the AOX respiration pathway and further affecting plant growth.

To demonstrate whether *PeAOX1b* is involved in tolerance to cold stress, the 17-day-old Arabidopsis seedlings were subjected to 4 °C for three days (Figure 7e), and leaf respiration tests showed that both total OCR and the proportion of the AOX pathway in total OCR increased significantly in transgenic lines compared to WT plants (Figure 7h,i). Further, cold-induced ELR rise and MDA accumulation were observed in all lines, with transgenic lines exhibiting significantly reduced ELR and MDA content compared to WT (Figure 7j,k). Furthermore, transgenic lines showed significantly higher Pro accumulation and SOD activity compared to WT under cold stress (Figure 7l,m). These results suggest that ectopic expression of *PeAOX1b* significantly enhances cold tolerance of Arabidopsis.

## 4. Discussion

Although considerable progress in understanding the fast-growth mechanisms of aboveground bamboo culm and the developmental mechanisms of bamboo rhizome bud has been made, the physiological changes and molecular regulation mechanism underlying the transition from initial growth of winter shoots to fast growth of spring shoots are still unknown. Previous studies evaluated the roles of phytohormones, carbohydrate metabolism, signal transduction, meristem development and transcriptional factors during rapid bamboo shoot growth [6,8,9,11,18,19]. However, as a central biological hub, mitochondrial biogenesis and the systematic energy regulation mechanism for shoot fast-growth initiation remain unknown. In this study, the mitochondria of Moso bamboo shoots were successfully isolated, and a comprehensive transcriptome and proteome analysis, for the first time, was conducted to investigate the regulation of bioenergetic processes for shoot fast-growth initiation in Moso bamboo.

### 4.1. Respiration and Energy Production Increased during the Transition from Winter to Spring Shoots

Sugars, the primary storage form for carbohydrates, are the main respiratory substrates and energy sources; therefore, sugar metabolism plays crucial roles in plant growth [47]. Plants respond quickly to endogenous sugar status during development and environmental changes in order to optimize their fitness and survival [48]. Traditionally, the long period of the shoot’s bud stage in the soil was regarded by bamboo farmers as the dormancy and nutrient accumulation period for further fast growth after the bamboo shoots emerged from the ground [49]. On the basis of our measurements, the starch and sucrose contents in winter shoots were indeed significantly higher than those in spring shoots (Figure 1a,b). This favors the traditional view that the development stage of underground shoots is the nutrient accumulation period and that shoot germination is accompanied by the large consumption of sugars, which has been confirmed by *Fargesia yunnanensis* shoot growth [50].

The consumption of carbohydrates, accompanied by enhanced energy metabolism, has been widely observed during diverse development processes in plants, such as rooting of cuttings [51], pollen-tube growth [52] and seed germination [21]. Studies on Moso bamboo have found that the energy required for rhizome bud growth mainly comes from carbohydrate metabolism pathways, and rhizome bud germination needs more energy and nutrients than early shoot stages [18]. In the culm rapid-growth stage, the energy metabolic activity varies in different internodes [53]. Compared to the start-division internode, *INV*, *SUT*, *PGAM* and *HK* genes showed higher expression levels in both rapid-division and rapid-elongation internodes. These are quintessential genes regulating carbohydrate metabolism for maintaining rapid growth of bamboo internodes. The development process from winter shoots to spring shoots is similar to seed germination, during which mitochondria are activated after seed imbibition, and respiration is enhanced accordingly, so as to rapidly produce energy to promote germination [54,55]. In the present study, 18 key enzyme genes responsible for starch and sucrose catabolism as well as carbohydrate metabolism were found upregulated during the spring shoot stage (Figure 2b–e). Moreover, based on high-resolution protein-separation technology and accurate quantitative analysis methods, our total proteome identified 12 significantly upregulated proteins related to energy metabolism (Table S8 and Table 1), which shows more diversity than data obtained using traditional quantitative methods such as two-dimensional electrophoresis [53]. The high consistency of energy gene upregulation at both the transcriptional and translational levels (Table 1) indicates that the systemic energy generation pathways were activated during the transition from winter to spring shoots. However, only a few genes involved in energy metabolism, such as *SUS*, *AMY*, *SUT* and *NST* were found upregulated during the transition between bud burst and rattling growth in *Dendrocalamus hamiltonii* (a subtropical bamboo) [13]. It is possible that respiratory activity and structural organization of functional mitochondrial respiratory supercomplexes vary with environmental factors and bamboo species, resulting in different levels of energy metabolism.

### 4.2. Different Ways of Energy Production Activated in Spring Shoots

Metabolic flexibility theory suggests that cells regulate methods of energy production according to their physiological needs [56]. During the developmental stages of rhizome bud and bamboo culm, the materials for tissue construction are mainly derived from stored nutrients and other autotrophic mechanism, as the photosynthetic system is not yet functional [18,53]. In the rhizome-bud-growth and early developmental stages of bamboo culm, the bamboo is covered with a sheath that generates an anaerobic environment. During these stages, stored nutrients are degraded into monosaccharides and participate in anaerobic respiration to produce energy, while aerobic respiration (e.g., TCA cycle, PPP) plays a minor role in energy generation [53]. In this study, glycolysis rate was obviously increased in spring shoots (Figure 1g), and more DEGs and DEPs were identified in EMP than in the TCA cycle and PPP (Figure 2c–e, Table 1), the key enzymes HK, PFK and GAPDH were especially upregulated in spring shoots, presumably resulting in an increase of the overall pool of ATP, PA and NADH (Figure 6). Anaerobic respiration will gradually lessen after the bamboo sheath is shed during the later developmental stage, and the TCA cycle will become the main source for energy [53].

In any case, OXPHOS is the main source of energy needed to sustain life activities, and 95% of ATP in organisms originates from this mechanism [26]. In this study, mitochondrial respiration was obviously enhanced in spring shoots (Figure 1g), and mitochondrial transcriptome analysis showed that all enzymes involved in OXPHOS (functioning by electron transfer chain) were upregulated at the spring shoot stage (Figure 3a), including NADH dehydrogenase (complex I), cytochrome reductase (complex III), cytochrome oxidase (complex IV) and ATP synthase (complex V). In the process of intracellular energy conversion, ATP synthase is a key enzyme existing in the mitochondrial inner membrane that synthesizes ATP in large quantities using the proton gradient and related membrane potential [43]. In mitochondrial proteome analysis, three ATP synthase proteins (PMA, ATPF1E and ATPeF1B) were also identified to be significantly upregulated (Table 2), suggesting that OXPHOS, as the main method of energy production, met the energy demand for fast-growth initiation of spring shoots.

### 4.3. Fission Activity and Number of Mitochondria Increased during the Transition from Winter to Spring Shoots

Mitochondria are created by fission of existing mitochondria [57], and their quantity and morphology are linked to metabolic requirements and cell energy as well as cell fate. To maintain basic intracellular metabolism and energy requirements, and to respond to various cell conditions, mitochondrial morphology and quantity need to be tightly controlled in cells [58]. An increase in the number of mitochondria usually reflects a rise in energy demand in cells. Previous studies have shown a dramatic increase in mitochondrial number in pollen during the early developmental stages [59], which relies on mitochondrial fission activity. This study showed that the mitochondrial number and ATP content in spring shoots increased significantly (Figure 1c–f), indicating that intensified mitochondrial fission produced sufficient numbers of mitochondria and energy to promote the initiation of rapid growth.

Several proteins are responsible for mitochondrial fission, among which dynamin and dynamin-related proteins play central roles [46]. In yeast, DNM1 is recruited to mitochondrial fission sites where it forms an oligomer that constricts mitochondria in order to facilitate their fission [60]. The *Arabidopsis thaliana* genome has two closely similar dynamin-related proteins (DRP), DRP3A and DRP3B, that are the functional orthologs of DNM1 [61,62]. In the *DRP3A DRP3B* double mutant, the mitochondria do not divide and form a massive, elongated network [61]. In addition, there are two plant-specific fission factors, ELM1 and PMDs. ELM1 interacts with DRP3A and perhaps DRP3B to recruit them to the mitochondrial fission sites [63]. Elongated mitochondria are also observed in the PMD1 mutant, but PMD1 and its paralog PMD2 do not physically interact with DRP3, suggesting that PMD1 facilitates mitochondrial proliferation in a DRP3-independent manner [64]. Herein, three candidate genes involved in mitochondrial fission (*DNM1*, *ELM1* and *PMD*) were obtained in the omics dataset (Figure 4). The finding that *DNM1* genes (but not *DRP3*) are expressed in Moso bamboo shoots indicates that dynamin-related proteins controlling mitochondrial division is specific among terrestrial plant species. Although the interaction between ELM1 and DRP3 has been confirmed, whether ELM1 interacts with DNM1 needs further study. Regardless, the upregulated expression of these three genes in spring shoots is conducive to enhancing the fission activity of mitochondria.

### 4.4. Mitochondrial Energy-Dissipating Systems Involved in the Adaptation of Bamboo Shoots to the Winter Environment

Previous studies have generally proven that AOX and UCP function in thermogenesis [65,66,67]. Their activity is generally low in common plant tissues but higher in the reproductive organs of some primitive seed plants from gymnosperm to angiosperm [68,69,70]. Their enhanced activity increases heat-energy release, thus promoting the emission of volatiles that may serve to attract pollinators [71,72] or preventing freeze damage in plants [73]. In the present study, mitochondrial transcriptome uncovered mitochondrial energy-dissipating pathways. Previously, Western blotting indicated AOX and UCP were detected in bamboo shoots of *Phyllostachys edulis* (whose new shoots develop from December to March in winter) but not in *Bambusa oldhamii* (whose new shoots develop from April to October in summer), suggesting that the adaptation of *Phyllostachys edulis* to a cooler environment may correlate with the higher energy-dissipating capacity of its AOX and UCP relative to *Bambusa oldhamii* [74].

Facing environmental pressure, plants regulate energy production and consumption by altering respiration metabolism so as to achieve a new homeostasis [75]. Under low-temperature stress, the level of mitochondrial reactive oxygen species (ROS) increases sharply. ROS in mitochondria mainly come from the mitochondrial ETC, and the generation of mitochondrial ROS generally depends on the reducing state of ETC components and the membrane potential [76]. AOX is believed to maintain the ETC components’ redox balance, thereby reducing mitochondrial ROS production and alleviating the damage caused to cell structures by membrane lipid peroxidation [77]. Further, AOX can promote signaling molecules (hydrogen peroxide, nitric oxide, superoxide and so on) to transmit information on the mitochondrial metabolism state to the nucleus, thereby regulating the synthesis of ROS scavengers such as Pro and SOD [78]. Our functional validation results suggest that under normal conditions, the decrease in total OCR and the COX-pathway proportion in *PeAOX1b* transgenic Arabidopsis reduced ATP production and thus delayed growth. However, under cold stress, the increase of total OCR and the AOX-pathway proportion in *PeAOX1b* transgenic plants could scavenge excess ROS through increasing Pro synthesis and SOD activity, thus better maintaining cell and mitochondrial homeostasis (Figure 7) and improving abiotic stress tolerance. Thus, the stronger respiratory activity of the AOX pathway and higher expression levels of *AOX* and *UCP* genes in winter Moso bamboo shoots (Figure 1j, Figure 3c, and Figure S7) may be involved in cold resistance, possibly by producing heat and /or stabilizing mitochondrial function.

## 5. Conclusions

In summary, the major findings deduced from physiological, cytological and omics analyses can be distilled into three main points:

1. Efficient energy-regulation mechanisms are essential for initiation of fast growth in Moso bamboo shoots; this entails a continuous process of carbohydrate oxidation consisting of starch and sucrose catabolism, EMP, the TCA cycle, the PPP and OXPHOS;
2. Mitochondrial fission increased starkly in accordance with the higher energy demand and cell division required for fast growth;
3. The revelation regarding the AOX pathway and UCP pathway and their enhanced roles in winter shoots reflects different adaptive heat requirements during shoot growth. These hypotheses suggest new avenues for further research into the mechanisms of bamboo fast growth and potentially for applications in edible-winter-shoot storage.

## 6. Patents

Lu, C.F., Wang, X.J., Cui, H.W., Geng, X., Yang, Y., Yu, T.Q. and Chen, Y.Z. One method for extraction, purification and identification of highly active mitochondria from Moso bamboo shoots. China, ZL 2019 1 0707028.1.

## Figures and Tables

**Table 1:** DEPs Related to Carbohydrate and Mitochondrial Energy Metabolism in Total Proteome.

Protein ID	Protein Description	Protein Name	Laber-Free		RNA-Seq	
			logFC	*p*-Value	logFC	*p*-Value
Carbohydrate Metabolism						
PH01001178G0320	40S ribosomal protein S3a	rpiA	−3.260	0.043	−0.289	0.026
PH01003555G0050	Phosphoglucomutase	pgm	−3.370	0.045	−3.823	0.000
PH01002199G0020	Isocitrate dehydrogenase	IDH3	1.203	0.049	0.901	0.000
PH01000533G0580	Aldehyde dehydrogenase	ALDH	2.862	0.008	2.611	0.000
PH01000669G0370	Hexokinase	HK	1.227	0.048	0.985	0.058
PH01001527G0160	Pyruvate kinase	PK	−1.930	0.017	−2.068	0.000
PH01001262G0280	Dehydrogenase E1 component domain containing protein	aceE	−3.307	0.005	−9.750	0.000
PH01002816G0060	Citrate synthase	CS	1.117	0.015	1.243	0.000
PH01000246G0090	6-phospho gluconate dehydrogenase decarboxylating	PGD	1.510	0.003	1.169	0.000
PH01000947G0420	Aldose1-epimerase	galM	12.761	0.000	1.949	0.000
Mitochondrial Energy Metabolism						
PH01004743G0090	2Fe-2S iron-sulfur cluster binding domain containing protein	NDUFS1	1.237	0.044	−0.467	0.288
PH01000376G0230	4Fe-4S binding domain containing protein	NDUFS8	1.888	0.028	−0.211	0.249
PH01002667G0100	NADH dehydrogenase 1 alpha subcomplex subunit 12	NDUFA12	11.493	0.000	0.039	0.855
PH01001330G0300	Solublein organic pyrophosphatase	PPA	10.091	0.005	0.789	0.000
PH01000704G0310	ATP synthase	ATPeF1B	1.206	0.020	1.084	0.000

Red indicates upregulated genes and proteins, and green indicates downregulated genes and proteins in spring shoot stage. Significant difference: *p* < 0.05.

**Table 2:** DEPs related to mitochondrial fission, carbohydrate and mitochondrial energy metabolism in mitochondrial proteome.

Protein ID	Protein Description	Protein Name	Laber-free		PRM	
			logFC	*p*-Value	logFC	*p*-Value
Carbohydrate Metabolism						
PH01000086G1010	Succinyl-CoA ligase subunit alpha-2	LSC1	10.721	0.000	0.571	0.050
PH01002199G0020	Isocitrate dehydrogenase	IDH3	2.838	0.011	0.863	0.032
PH01000713G0340	ATP-citrate synthase subunit1	ACLY	−1.534	0.000	1.271	0.002
PH01000947G0420	Aldose1-epimerase	galM	7.663	0.018	NA	NA
PH01001262G0210	Pyrophosphate-fructose6-phosphate1-phospho transferase subunit beta	pfp	5.690	0.008	1.958	0.009
PH01003425G0070	Glyceraldehyde-3-phosphate dehydrogenase	GAPDH	−1.366	0.000	1.556	0.000
PH01002248G0250	Pyruvate phosphate dikinase	ppdK	3.962	0.005	1.923	0.100
PH01000031G1210	Fructose-bisphospate aldolase isozyme	ALDO	3.264	0.000	1.642	0.007
PH01003555G0050	Phosphorglucomutase	pgm	−9.163	0.001	−1.459	0.239
PH01001178G0320	40S ribosomal protein S3a	rpiA	−1.871	0.034	−1.702	0.000
PH01001444G0350	Aldehyde dehydrogenase	gapN	2.787	0.003	−0.075	0.855
PH01003304G0200	Hexokinase	HK	2.162	0.019	0.609	0.119
PH01000891G0110	Aldehyde dehydrogenase	ALDH	10.936	0.002	NA	NA
Mitochondrial Energy Metabolism						
PH01000322G0030	Pyruvate decarboxylase isozyme 2	PDC	2.396	0.020	1.601	0.138
PH01000597G0060	H^+^-transporting ATPase	PMA	2.193	0.036	0.825	0.023
PH01000965G0370	F-type H^+^-transporting ATPase subunit epsilon	ATPF1E	9.684	0.015	2.376	0.025
PH01001308G0210	Solublein organic pyrophosphatase	PPA	4.246	0.000	−0.389	0.562
PH01001580G0330	NADH dehydrogenase 1 alpha subcomplex subunit 9	NDUFA9	2.149	0.023	1.365	0.000
PH01000276G0370	LYR motif containing protein	NDUFB9	2.022	0.033	NA	NA
PH01003254G0190	Ribosomal protein L51	NDUFA2	1.977	0.025	0.723	0.101
PH01002851G0110	2Fe-2Siron-sulfur cluster binding domain containing protein	NDUFS1	1.644	0.045	1.034	0.006
PH01002719G0170	Cytochrome b-c1 complex subunit 7	fbcH	1.647	0.048	0.875	0.000
PH01000704G0310	F-type H^+^-transporting ATPase subunit beta	ATPeF1B	5.056	0.004	1.749	0.019
Mitochondrial Fission						
PH01001466G0130	Dynamin 1	Dnm1	12.187	0.000	NA	NA

Yellow indicates upregulated genes and proteins, and blue indicates downregulated genes and proteins in spring shoot stage. NA indicated that PRM detection was not performed. Significant difference: *p* < 0.05.

## Data Availability

The RNA-seq data have been deposited to the NCBI Short Read Archive (accession number: PRJNA681325, https://www.ncbi.nlm.nih.gov/sra/PRJNA681325 (accessed on 6 July 2021)). The mass spectrometry proteomics data are available in the ProteomeXchange Consortium (accession number: PXD028178, http://proteomecentral.proteomexchange.org (accessed on 31 December 2021)).

## Supplementary Materials

The following supporting information can be downloaded at: https://www.mdpi.com/article/10.3390/cells11071240/s1, Method S1: Measurements of mitochondrial respiration rate and glycolysis rate; Method S2: Mitochondrial isolation; Method S3: Mitochondrial activity detection; Method S4: Mitochondrial count; Method S5: Quantitative verification of targeted protein by PRM; Method S6: Detection of electrolyte leakage rate; Figure S1: Moso bamboo shoots in different development stages; Figure S2: Activity and purity identification of mitochondria isolated from Moso bamboo shoots; Figure S3: RT-PCR identify and expression analysis of PeAOX1b transgenic Arabidopsis T3 homozygous lines; Figure S4: Volcano plot of differentially expressed genes; Figure S5: GO functional enrichment analysis of DEGs in total transcriptome; Figure S6: KEGG functional enrichment analysis of DEGs in total transcriptome; Figure S7: Relative expression level of carbohydrate metabolism genes at two time points of Moso bamboo shoots development detected by RNA-seq and RT-qPCR; Figure S8: Expression level of co-expressed mitochondrial genes in total and mitochondrial transcriptome; Figure S9: Relative expression level of energy metabolism genes at two time points of Moso bamboo mitochondria detected by RNA-seq and RT-qPCR; Figure S10: Overview of label-free quantitative analysis of total and mitochondrial proteome; Figure S11: GO functional enrichment analysis of DEPs in total proteome; Figure S12: KEGG functional enrichment analysis of DEPs in total proteome; Figure S13: GO functional enrichment analysis of DEPs in mitochondrial proteome; Figure S14: KEGG functional enrichment analysis of DEPs in mitochondrial proteome; Figure S15: Expression analysis of the top 20 differentially expressed correlations (DECs) with the highest significance; Figure S16: KEGG functional enrichment analysis of the differentially expressed correlations (DECs); Table S1: Primers of organelle specific genes for mitochondrial purity identification; Table S2: Primers used in RT-qPCR; Table S3: Respiratory ratio of winter and spring shoot before and after the addition of different inhibitors; Table S4: Summary of sequencing data of total transcriptome and alignment information of clean reads; Table S5: DEGs related to starch and sucrose metabolism in total transcriptome; Table S6: Summary of sequencing data of mitochondrial transcriptome and alignment information of clean reads; Table S7: Comparison of protein-coding gene content between Phyllostachys edulis mitochondrial transcriptome and other grasses mitochondrial genomes; Table S8: DEPs related to starch and sucrose metabolism in total proteome.
