# Supplementary material for: Total and Mitochondrial Transcriptomic and Proteomic Insights into Regulation of Bioenergetic Processes for Shoot Fast-Growth Initiation in Moso Bamboo
Source: Cells. 2022 Apr 6;11(7):1240. doi: 10.3390/cells11071240 (PMC8997719; doi:10.3390/cells11071240)
Supplement: Supplementary file 1 [file cells-11-01240-s001.zip › cells-1656330-supplementary.pdf]

Total and mitochondrial transcriptomic and proteomic insights into regulation of bioenergetic processes for shoot fast growth initiation in Moso bamboo

Xiaojing Wang [1], Xin Geng [1], Lilin Yang [2], Yuzhen Chen [1], Zhiheng Zhao [1], Weijia Shi [1], Lan Kang [1], Ruihua Wu [1], Cunfu Lu [1*], and Jian Gao [3*]

[1] College of Biological Sciences and Technology, National Engineering Laboratory for Tree Breeding, Beijing Forestry University, Beijing, China, 100083

[2] College of Agriculture and Forestry Engineering and Planning, Tongren University, Tongren, China, 554300

[3] International Center for Bamboo and Rattan, Key Laboratory of Bamboo and Rattan Science and Technology, State Forestry and Grassland Administration, Beijing, China, 100102

Supplementary Methods

Method S1. Measurements of mitochondrial respiration rate and glycolysis rate

The single tissue disc (2.5-mm-diameter and 200-μm-thickness) used for testing was freshly cut from the middle part of bamboo shoots. The respiration buffer (5 mM KH_2PO_4, 10 mM TES, 10 Mm NaCl, 2 mM $MgSO_4$, pH 7.2) was added to the wells. Where indicated, inhibitors were added to the medium with a final concentration of 50 mM NaN_3 or 5 mM SHAM. The OCR and ECAR of the single shoots tissue disc were recorded by Seahorse XF Acquisition and Analysis Software (Wave 2.6.1, Seahorse Bioscience).

Method S2. Mitochondrial isolation

The outer leaf sheaths of bamboo shoots were carefully peeled off manually prior to treatment. About 4 cm was removed from the cut end of each shoot with a sharp kitchen knife. Plant tissues were homogenized at 4 °C in extraction buffer (1.25 M NaCl, 0.3 M Sucrose, 50 mM Tris-HCl, 5 mM EDTA, 2 mM EGTA, 0.5% [w/v] BSA, 0.5% [w/v] PVP-40, and 15 mM thioglycol, pH 8.0) with a homogenizer (200 mL of buffer for 150 g of plant tissues, ten bursts

of 5 s, speed 18000 r min^{-1}). The nuclei from this homogenate were sedimented (1500 g for 5 min at 4 °C, two times), chloroplast was sedimented from the supernatant by centrifugation (6,000 g for 10 min at 4 °C, two times). Mitochondria were sedimented from the supernatant by centrifugation (15,000 g for 15 min at 4 °C). The pellet was resuspended in wash buffer (0.35 M Sucrose, 25 mM EDTA, and 50 mM Tris-HCl, pH 8.0) and layered on a discontinuous sucrose gradient (from bottom to top: 40% and 23% [w/v]). The gradient was centrifuged at 20,000 g for 45 min at 4 °C. The mitochondrial fraction was collected from the interface between the two sucrose cushions, diluted with wash buffer and washed once. The purified mitochondrial pellet was finally resuspended in wash buffer.

Method S3. Mitochondrial activity detection

The purified mitochondrial suspension and 0.4% [w/v] Janus green B were mixed (1:1, v/v). After staining at room temperature for 30 minutes, mitochondria were observed with DMIL LED inverted microscope (Leica, Frankfurt, Germany). The active mitochondria showed a distinct blue-green color (Figure S2A, S2B). The purified mitochondrial suspension and 1mM Rhodamine 123 were mixed (1:1, v/v) at 30 °C in dark for 5 min and observed under a laser scanning confocal microscope (Nikon, Tokyo, Japan) (excitation wavelength/emission wavelength, 507/529 nm). The active mitochondria emited a distinct yellow-green fluorescence (Figure S2C, S2D).

Method S4. Mitochondrial count

Here, the purified mitochondrial fractions were prepared respectively as mentioned above using the same quality (150 g) of tissues from the middle part of winter shoot and spring shoot, then resuspended in 2 ml wash buffer and labeled with Rhodamine 123 (1mM; 2ml), finally counted and analyzed by CytoFLEX flow cytometry (Beckman Coulter, USA) and CytExpert software (Beckman Coulter).

Method S5. Quantitative verification of targeted protein by PRM

Protein extraction and tryptic digestion were performed in the same way as in the Laber-free experiment. MS data acquisition was first performed in DDA mode to obtain MS/MS spectra for the 40 most abundant precursor ions following each survey MS1 scan in each cycle. Protein Pilot software was used to identify proteins, and the database searching results were brought into Skyline software for spectra library building. Target proteins for PRM validation were

imported to the software Skyline, and the peptides for protein quantification were selected according to the ion signals in spectra library. A list of associated peptides containing m/z values and retention times was exported from Skyline, and imported to MS control software Analyst for PRM acquisition method construction. PRM method was run against the mitochondrial samples, evaluated and refined to develop the highest quality assay. Data collection of each sample was performed using the final PRM acquisition method on the mass spectrometer, where each precursor ion was selected by the quadrupole, fragmented, and then all fragment ions were quantified in the mass analyzer. Data processing was done in Skyline, and the quantification results were manually inspected for each peptide of the targeted proteins. All proteins with a *P*-value below 0.05 and a fold change larger than 1.5 were considered significant.

Method S6. Detection of electrolyte leakage rate

Briefly, Arabidopsis seedlings (100 mg) were collected and soaked in 8 ml distilled water at room temperature for 10 h, the initial conductivity (C1) was determined using a conductivity meter. Then sample was boiled for 30 minutes, and the final conductivity (C2) was measured after sample cooling to room temperature. The electrolyte leakage rate was calculated according to ELR (%) = C1/C2×100.

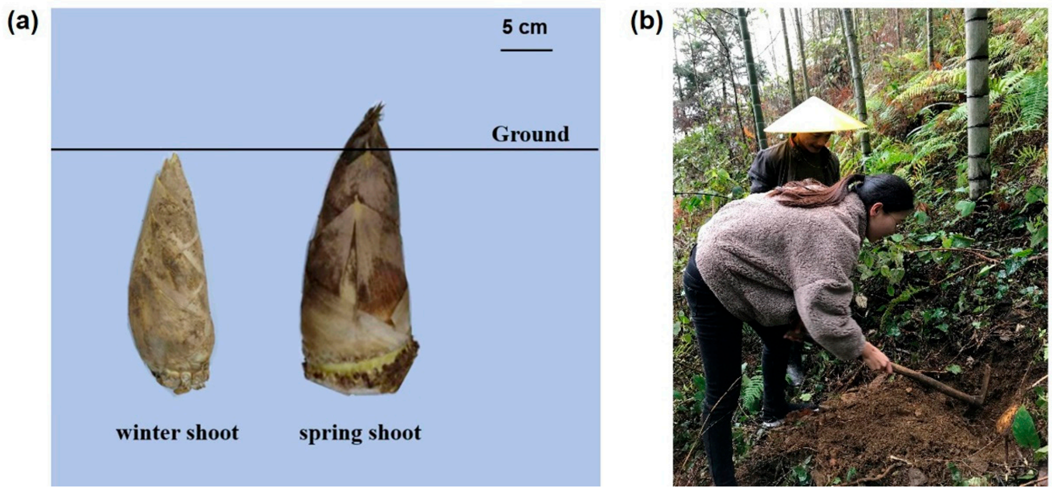

Figure S1. Moso bamboo shoots in different development stages. (a) Winter and spring shoots. (b) Sampling in the Moso bamboo forest.

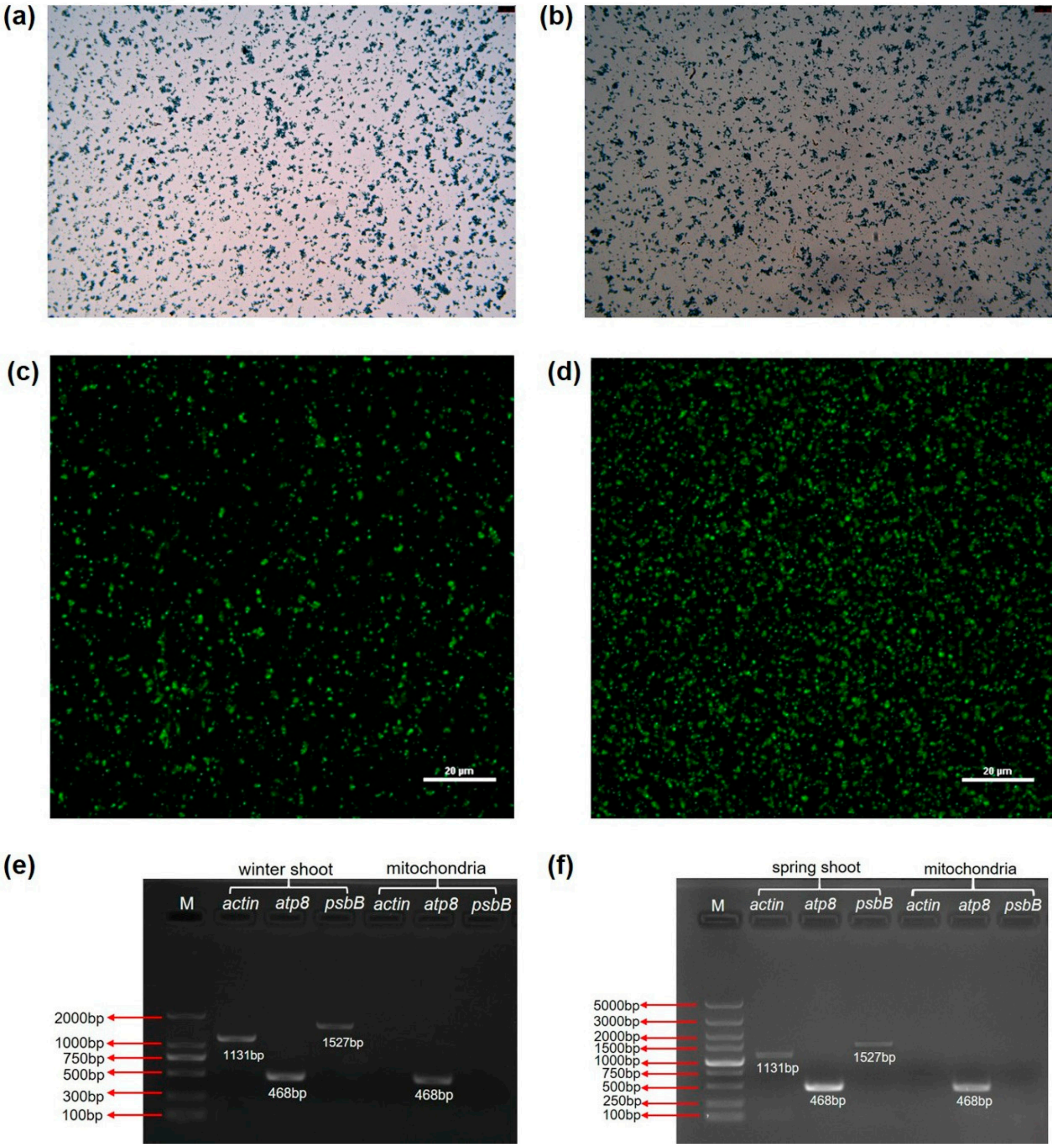

Figure S2. Activity and purity identification of mitochondria isolated from Moso bamboo shoots. The activated mitochondria from winter shoot (a) and spring shoot (b) stained with Janus green B were observed under light microscope, and the length of the black bar in the upper right corner is 100 μm. The activated mitochondria from winter shoot (c) and spring shoot (d) stained with Rhodamine 123 were observed under confocal laser microscope, and the white line at the bottom right represents 20 μm. Mitochondrial purity identification of winter shoot (e) and spring shoot (f) by PCR amplification of organelle specific genes.

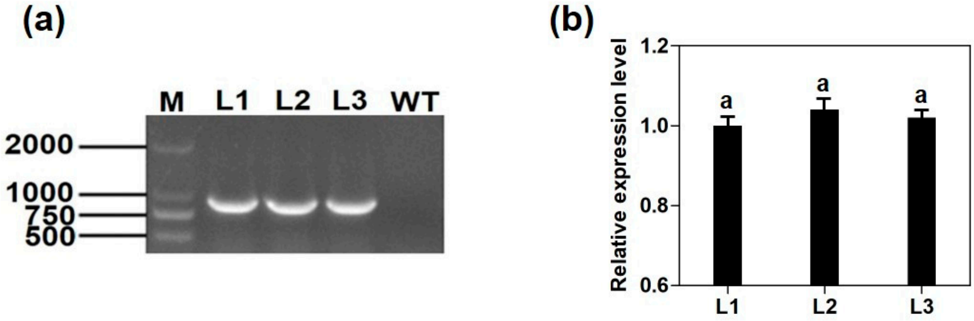

Figure S3. RT-PCR identify and expression analysis of *PeAOX1b* transgenic Arabidopsis T3 homozygous lines. (a) RT-PCR identify of *PeAOX1b* transgenic Arabidopsis T3 homozygous lines. (b) The relative expression level of three transgenic Arabidopsis lines. M: 2000 bp Maker; WT: Wild type Arabidopsis; L1-L3: Transgenic Arabidopsis. Significant difference criteria: $P < 0.05$.

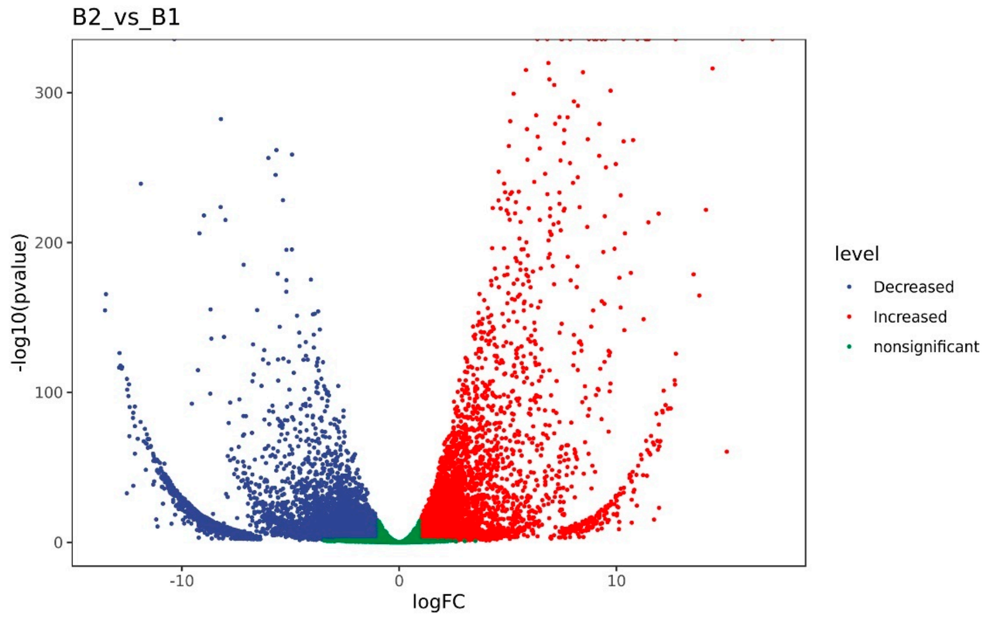

Figure S4. Volcano plot of differentially expressed genes. X-axis and Y-axis present threshold value in log transform. Each dot is a DEG. Dots in red and blue mean significantly up-regulated and down-regulated DEGs which passed screening threshold and green dots are non-significant DEGs. Abbreviation: B1, winter shoot; B2, spring shoot

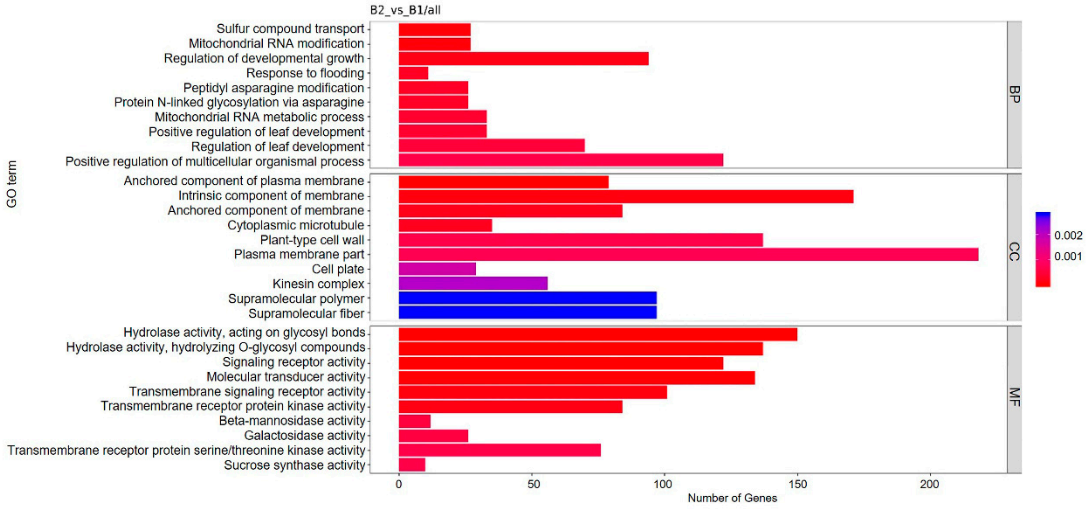

Figure S5. GO functional enrichment analysis of DEGs in total transcriptome. X axis means number of DEGs. Y axis represents GO terms. All GO terms are grouped in to three ontologies: BP (biological process), CC (cellular component), MF (molecular function). Gradient color barcode at the right indicates *p*-value, and less *p*-value means greater intensiveness. We just display the top 10 of enriched GO terms in each group. Abbreviation: B1, winter shoot; B2, spring shoot.

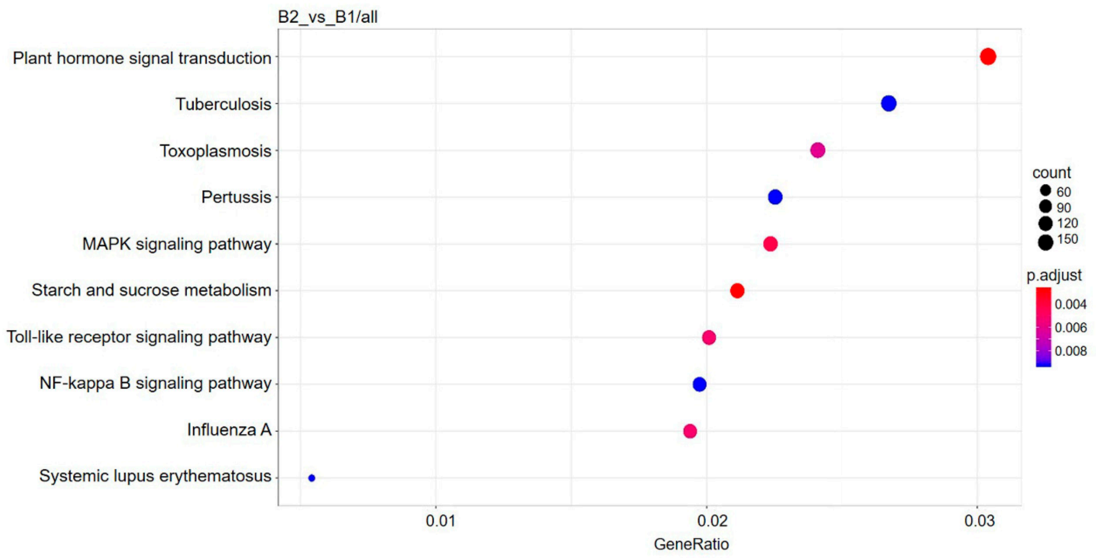

Figure S6. KEGG functional enrichment analysis of DEGs in total transcriptome. X axis means GeneRatio. Y axis represents KEGG pathway terms. GeneRatio is the ratio of DEG numbers annoted in this pathway term to all gene numbers annoted in this pathway term. Greater GeneRatio means greater intensiveness. The number of DEGs is represented by the size of the circle, and the larger circle means the more DEGs. P.adjust is corrected *p*-value ranging from 0~1, and less p.adjust means greater intensiveness. We just display the top 10 of enriched pathway terms. Abbreviation: B1, winter shoot; B2, spring shoot.

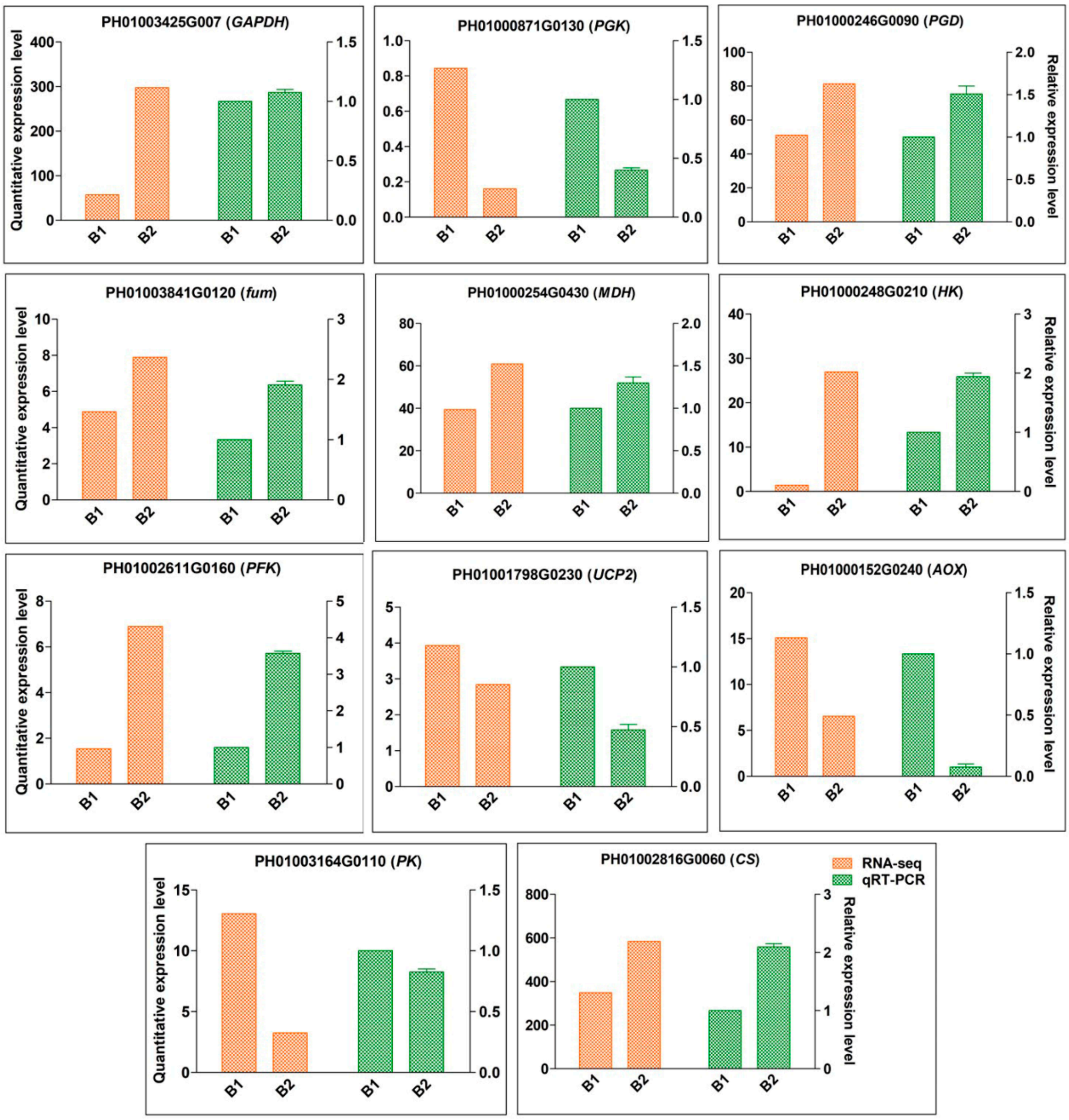

Figure S7. Relative expression level of carbohydrate metabolism genes at two time points of Moso bamboo shoots development detected by RNA-seq and RT-qPCR. Abbreviation: B1, winter shoot; B2, spring shoot.

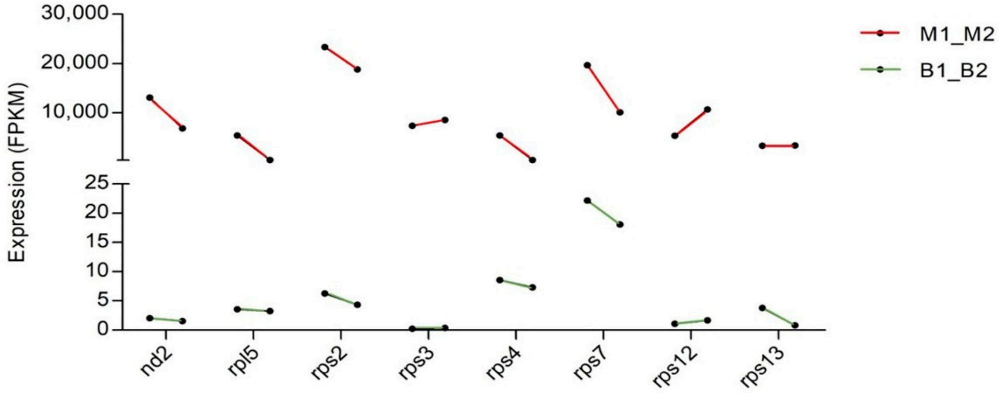

Figure S8. Expression level of co-expressed mitochondrial genes in total and mitochondrial transcriptome. Abbreviations: M1, winter shoot mitochondria; M2, spring shoot mitochondria; B1, winter shoot; B2, spring shoot

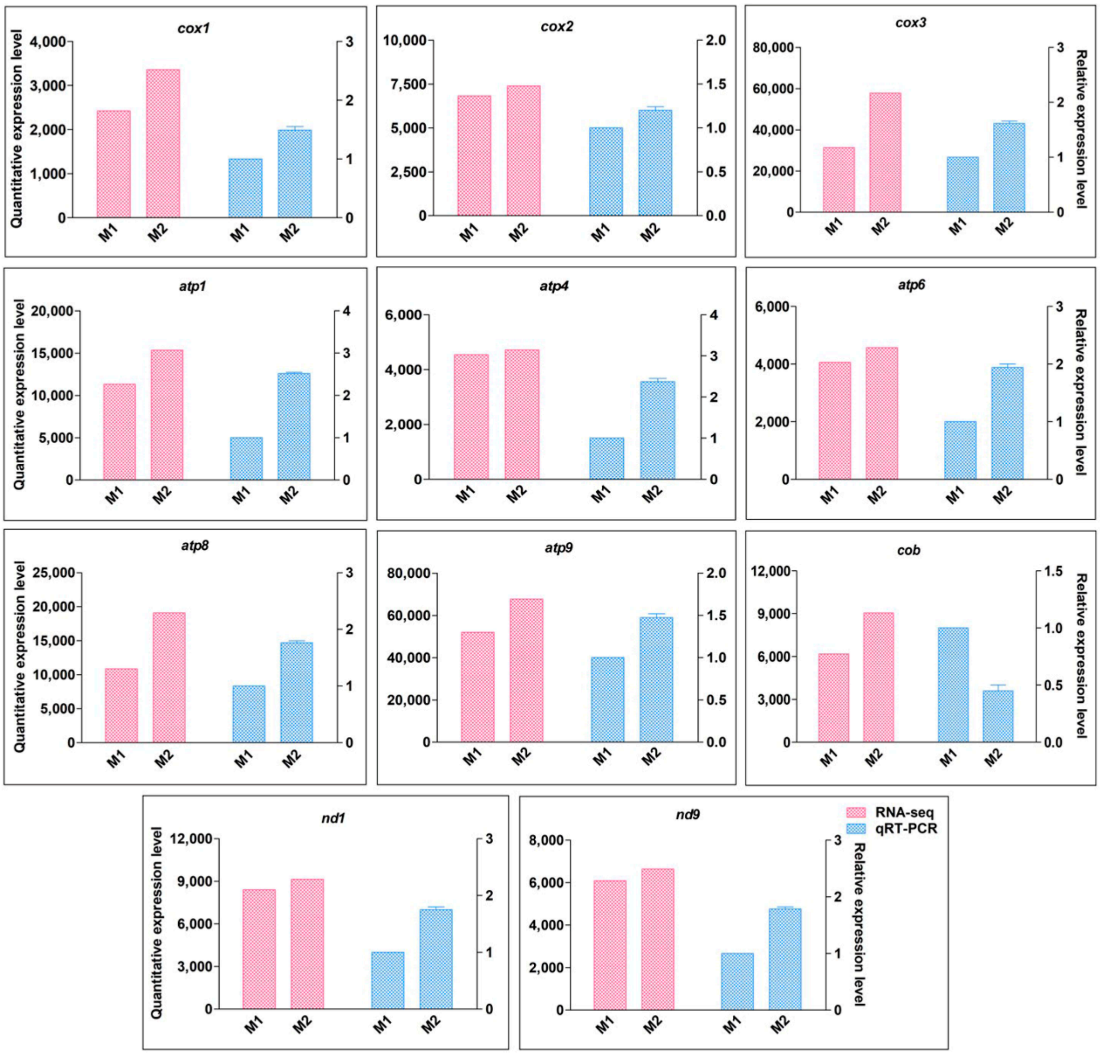

Figure S9. Relative expression level of energy metabolism genes at two time points of Moso bamboo mitochondria detected by RNA-seq and RT-qPCR. Abbreviation: M1, winter shoot mitochondria; M2, spring shoot mitochondria.

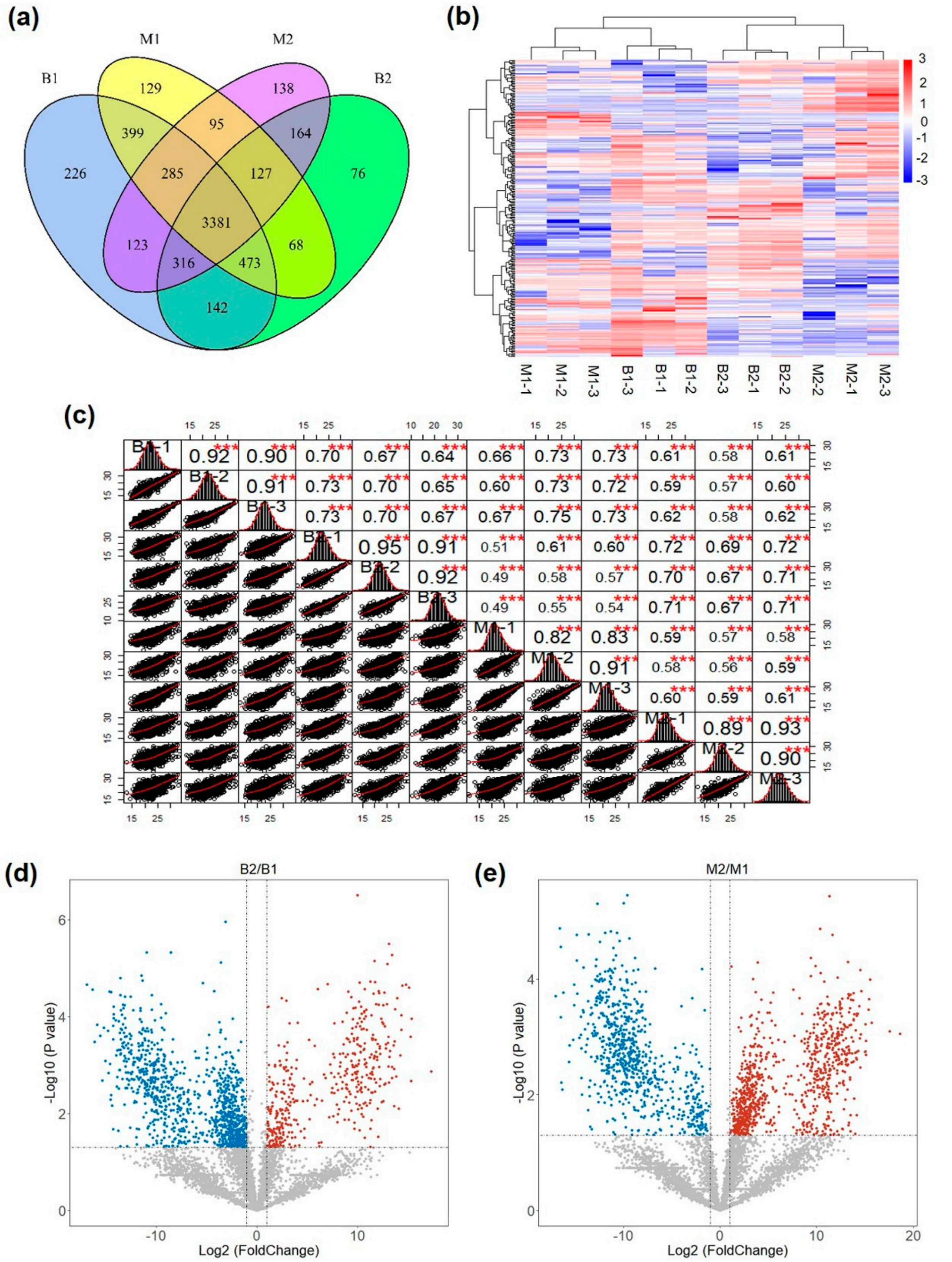

Figure S10. Overview of Label-free quantitative analysis of total and mitochondrial proteome. (a) The number of proteins identified in different samples. (b) The global expression profile of proteins identified in different samples. The color scale (−3.0 to 3.0) represents the Z-score calculated. Red represents up-regulated expression and blue represents down-regulated expression. (c) Quantitative correlation analysis between samples. (d) Volcano plot of DEPs in total proteome. (e) Volcano plot of DEPs in mitochondrial proteome. For volcano plot, X-axis

and Y-axis present threshold value in log transform. Each dot is a DEG. Dots in red and blue mean significantly up-regulated and down-regulated DEGs which passed screening threshold and gray dots are non-significant DEGs. Abbreviation: B1, winter shoot; B2, spring shoot; M1, winter shoot mitochondria; M2, spring shoot mitochondria.

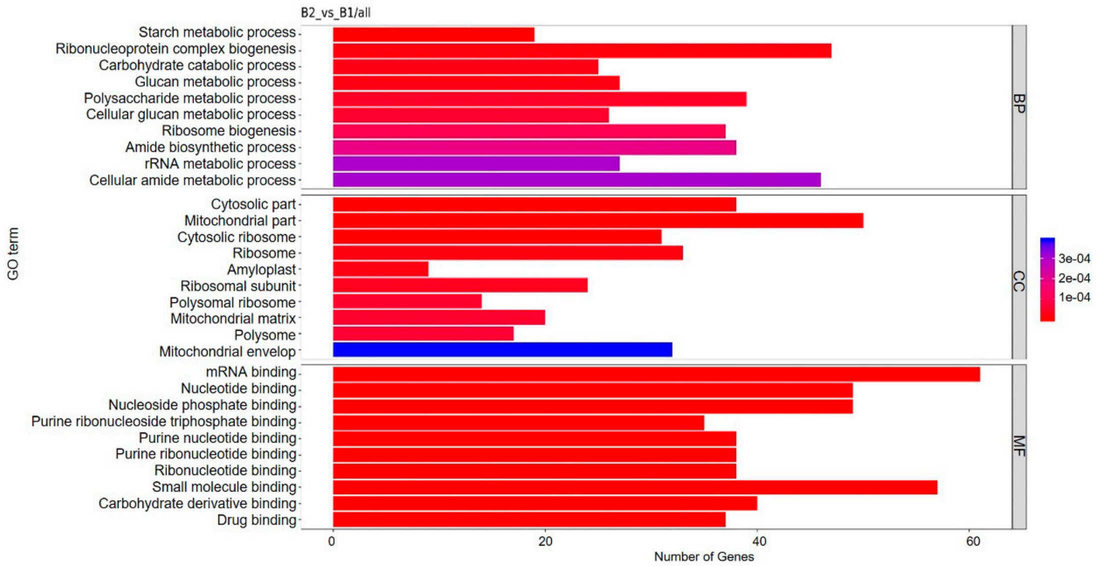

Figure S11. GO functional enrichment analysis of DEPs in total proteome. X axis means number of DEPs. Y axis represents GO terms. All GO terms are grouped in to three ontologies: BP (biological process), CC (cellular component), MF (molecular function). Gradient color barcode at the right indicates *p*-value, and less *p*-value means greater intensiveness. We just display the top 10 of enriched GO terms in each group. Abbreviation: B1, winter shoot; B2, spring shoot.

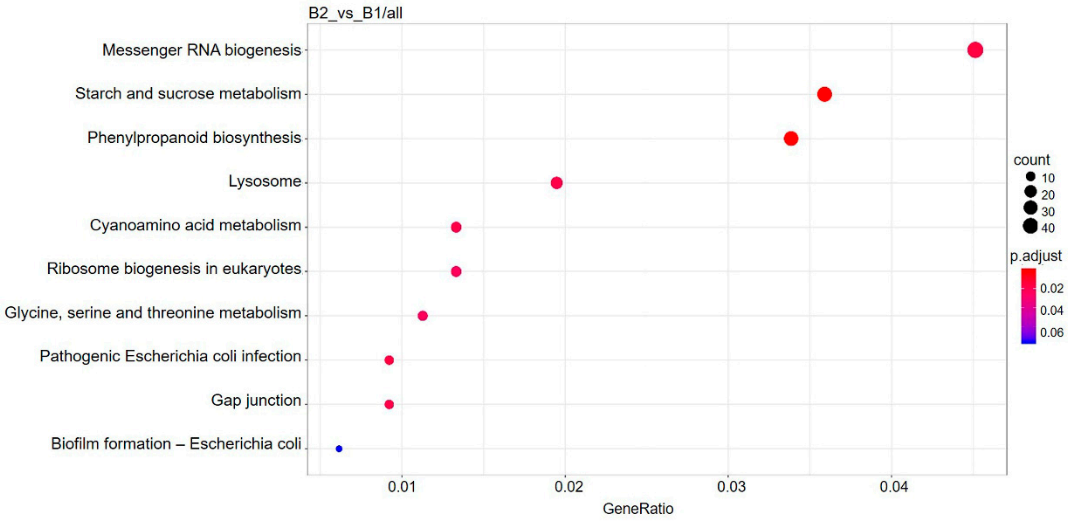

Figure S12. KEGG functional enrichment analysis of DEPs in total proteome. X axis means GeneRatio. Y axis represents KEGG pathway terms. GeneRatio is the ratio of DEP numbers annoted in this pathway term to all protein numbers annoted in this pathway term. Greater GeneRatio means greater intensiveness. The number of DEPs is represented by the size of the circle, and the larger circle means the more DEPs. P.adjust is corrected *p*-value ranging from 0~1, and less p.adjust means greater intensiveness. We just display the top 10 of enriched pathway terms. Abbreviation: B1, winter shoot; B2, spring shoot.

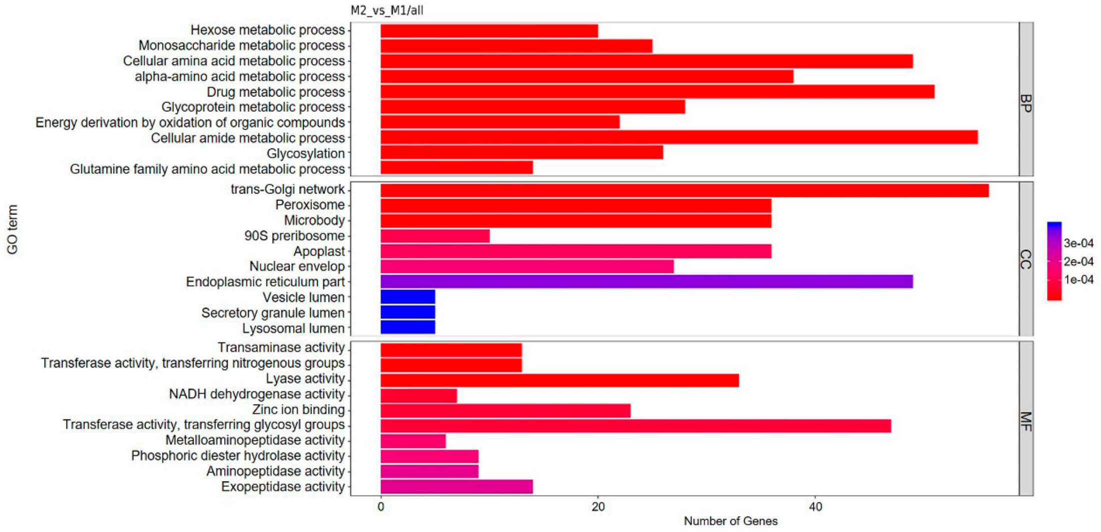

Figure S13. GO functional enrichment analysis of DEPs in mitochondrial proteome. X axis means number of DEPs. Y axis represents GO terms. All GO terms are grouped in to three ontologies: BP (biological process), CC (cellular component), MF (molecular function). Gradient color barcode at the right indicates *p*-value, and less *p*-value means greater intensiveness. We just display the top 10 of enriched GO terms in each group. Abbreviation: M1, winter shoot mitochondria; M2, spring shoot mitochondria.

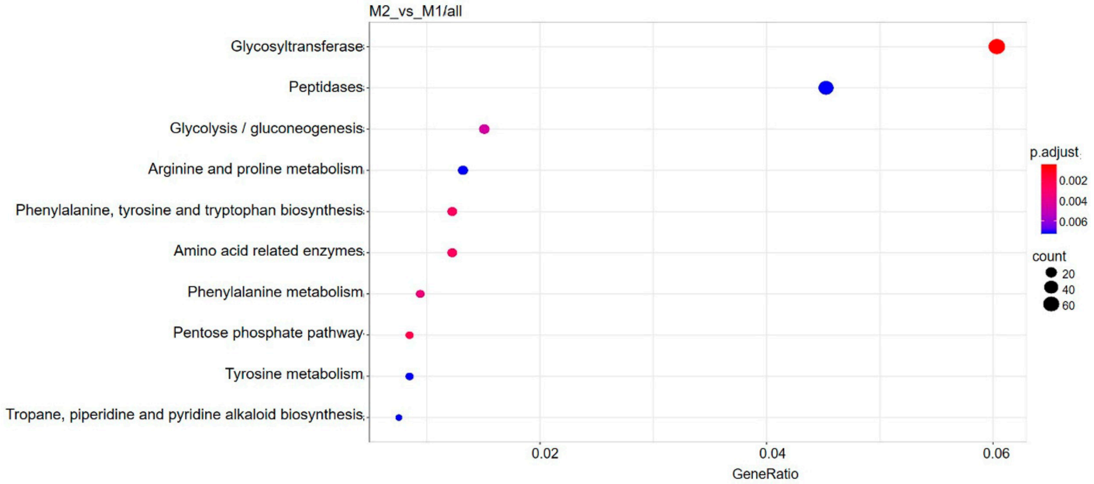

Figure S14. KEGG functional enrichment analysis of DEPs in mitochondrial proteome. X axis means GeneRatio. Y axis represents KEGG pathway terms. GeneRatio is the ratio of DEP numbers annoted in this pathway term to all protein numbers annoted in this pathway term. Greater GeneRatio means greater intensiveness. The number of DEPs is represented by the size of the circle, and the larger circle means the more DEPs. P.adjust is corrected *p*-value ranging from 0~1, and less p.adjust means greater intensiveness. We just display the top 10 of enriched pathway terms. Abbreviation: M1, winter shoot mitochondria; M2, spring shoot mitochondria.

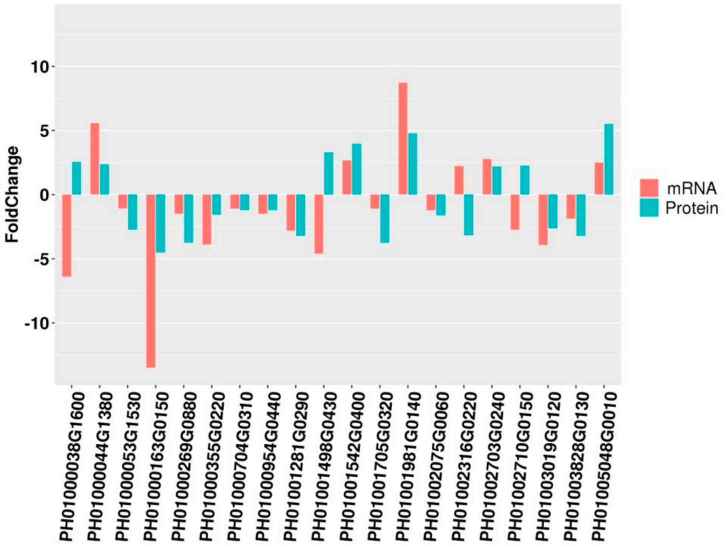

Figure S15. Expression analysis of the top 20 differentially expressed correlations (DECs) with the highest significance.

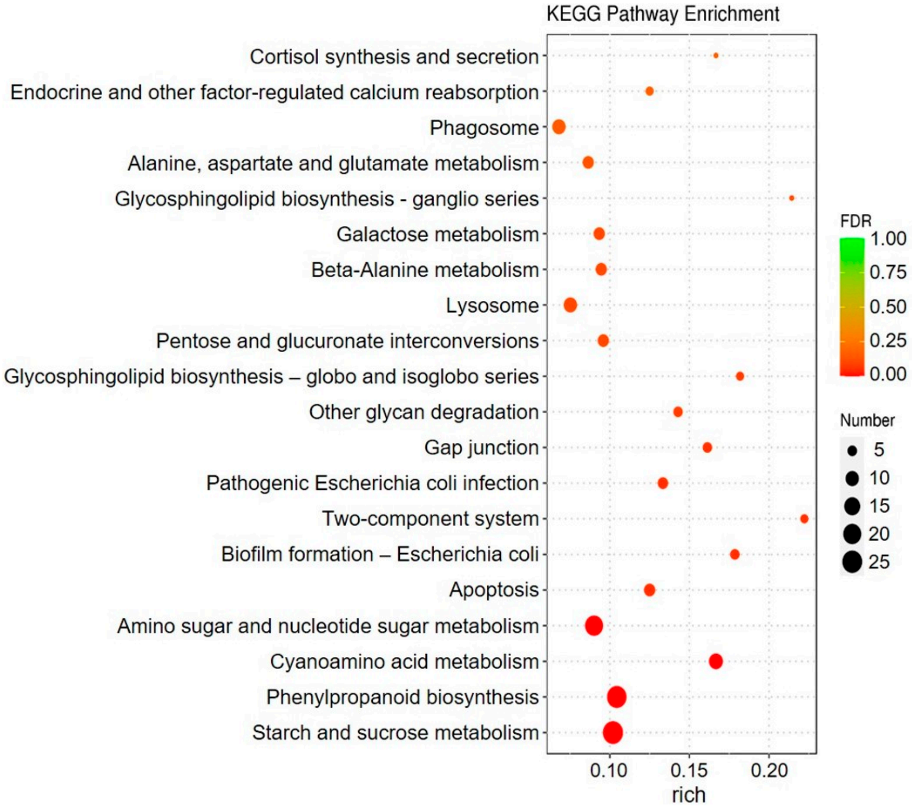

Figure S16. KEGG functional enrichment analysis of the differentially expressed correlations (DECs). X axis indicates rich factor. Y axis means KEGG pathway terms. Rich factor is the ratio of DEC numbers annoted in this term to all gene numbers annoted in this term. Greater rich factor represents greater intensiveness. The number of DECs is represented by the size of the circle, and the larger circle means the more DECs. FDR is false discovery rate ranging from 0~1, and less FDR means greater intensiveness. We just display the top 20 of enriched terms.

Table S1. Primers of organelle specific genes for mitochondrial purity identification.

Unigene	Forward Primer (5’ - 3’)	Reverse primer 5’ - 3’
Pe-actin	ATGGCTGAAGAGGATATCCAG	CTAGAAACACTTCATATGGAC
Pe-atp8	ATGCCTCAACTTGATAAATT	TTAGATTATGCTTCCTTGCC
Pe-psbB	ATGGGTTTGCCTTGGTATCGT	TCAGACTGCCTGTCTCCTTGT

Table S2. Primers used in RT-qPCR.

Target unigene	Forward Primer (5' - 3')	Reverse primer (5' - 3')
**For bamboo shoots samples**		
actin	GGTGTGAGCCATACTGTGCCCAT	TTTCCCGTTCAGCAGAGGTTGTG
TIP41	CATTCGCTAGCTTGTCTGCG	CACGGGAAAGGTGTGTTAGC
GAPDH	CTCAAGCAAGGACTGGAGGG	CGGTCAGGTCAACGACAGAA
PGK	CCCACCATCAAGCACCTCAT	ACCTGTACGCCAAGAAGCTC
PGD	GGGATGGGTGTTTCTGGAGG	ATGGGCCACTATCAGGGACT
Fum	ATGACATTCGCTTGCTGGGA	TGCCCATTCGAACCACCTAC
MDH	GACCTATTCAACATCAACGCCG	GGTCACACCAAACAGCTTCTTC
HK	GCCATGGAGAAGCAAGGTCT	CCTTTTCGACATAAGCGGCG
PFK	ACCAGCGTTGTTGGCATAGA	GTCCTCCCCTTGAAGTTCCG
UCP2	TGGACCGAACATTGCACGTA	GCACCCAAACCAGCAAAGAG
AOX	AATGATGCGCATGAAAGGCC	AGCAGCTCCATTGGCATCAA
PK	GGTGTGTGGTCTAAGCCGAA	GGTGTGATGCATGGTCTCCA
CS	GTTATCTCAACACCGCCCCA	TTGTGAACTCCCAGTCTGCC
**For mitochondrial samples**		
rps13	GAAGAGGGGAGAACGAGCAG	TGAGTTCGTTGACCGCGTAA
ccmFC	TTACTTCCATGGTCGTGCCC	CAAACAAGCACCACTCGACG
cox1	TGGGATTCGTCGTTTCTTCGT	AAGTATGAAAGGCTGGAGGGC
cox2	CTTTGTGATGCTGCGGAACC	AATGCCATAAAGCGCGAACC
cox3	ATTCTTCTTTGGCACCTACGGT	GCGTAAACAGCTCGTTTTTCCT
atp1	AGATCGGTCGAGTGGTCTCA	TCCAGTGCGCTTGACAAGAT
atp4	TGCGAAAAGACAGTGCAAGC	AGCTTTGAACGTAGACCCGG
atp6	GCAGCAAAATGGGGCTTCAA	GACCAAACCGAGAGTGAGCA
atp8	GGGAACAAGATCCGGAGCAA	CCATTCCTCGTGAGCCACTT
atp9	ATCAATAGGTGCCGGAGCTG	CAATGATGGATTTCGCGCCA
cob	TGTTCGGTGTCTCGGAGTTG	AGAAACCACCCCAAAGCCAA
nd1	AGCATTACGATCTGCAGCTCA	CCAATACGGGGAACAAGGGAA
nd9	AATCATCCGGATTTACGCCGTA	TGGGTCATCTCAATGGGTTCAG

Table S3. Respiratory ratio of winter and spring shoot before and after the addition of different inhibitors.

Time (min)	Sequential additions	OCR (pmol min^{-1})	
		Winter shoot	Spring shoot
78.36	Before additions	143.25±32.89 ^{a}	198.18±20.98 ^{a}
91.12	NaN_3	92.03±23.51 ^{b}	134.48±29.09 ^{b}
116.55	SHAM	60.82±18.59 ^{c}	108.11±20.22 ^{c}

OCR, oxygen consumption rate; NaN_3, cytochrome c oxidase (COX) respiratory pathway inhibitor sodium azide; SHAM, alternative oxidase (AOX) respiratory pathway inhibitor salicylhydroxamic acid. The mean and standard deviation (SD) were calculated from three biological replicates. The letters a, b, c in the table indicate significant differences between the same column of data ($P < 0.05$).

Table S4. Summary of sequencing data of total transcriptome and alignment information of clean reads.

Sample	Raw data base (bp)	Clean data base (bp)	Raw data reads	Clean data reads	Total clean reads	Total mapping ratio (%)	Uniquely mapping ratio (%)
B1_1	5,725,652,100	4,978,328,916	38,171,014	36,754,224	35,064,600	90.21%	86.93%
B1_2	7,594,477,200	6,580,675,249	50,629,848	48,447,218	40,457,496	86.34%	83.27%
B1_3	9,291,500,100	8,042,215,023	61,943,334	59,235,322	49,620,884	86.21%	83.05%
B2_1	4,074,935,100	3,553,398,487	27,166,234	26,178,356	23,980,918	90.13%	86.92%
B2_2	7,582,074,600	6,473,773,868	50,547,164	47,899,098	36,381,450	82.85%	78.86%
B2_3	8,814,375,900	7,580,801,613	58,762,506	55,965,190	42,162,828	83.51%	79.56%
Mean value	7,180,502,500	6,201,532,193	47,870,017	45,746,568	37,944,696	86.54%	83.10%

Table S5. DEGs related to starch and sucrose metabolism in total transcriptome.

Gene ID	Level	Log FC	Gene ID	Level	Log FC
**Invertase (INV)**			**Amylase (AMY)**		
PH01000019G0910	Up regulated	4.77303	PH01001272G0030	Up regulated	2.67567
PH01000019G0990	Up regulated	4.04906	PH01000378G0910	Up regulated	2.43842
PH01000019G0920	Up regulated	4.46995	PH01001911G0350	Up regulated	1.71811
PH01001102G0470	Up regulated	1.95003	PH01000378G0940	Up regulated	1.00011
**Fructokinase (scrK)**			PH01000010G1130	Up regulated	4.08091
PH01000235G0540	Up regulated	2.35270	PH01000560G0630	Up regulated	10.11074
**Glucose-6-phosphate isomerase (GPI)**			PH01000171G0500	Up regulated	3.73910
PH01000589G0720	Up regulated	1.64176	PH01000585G0580	Up regulated	1.21965
**Sucrose synthase (SUS)**			**Isoamylase (ISA)**		
PH01008776G0020	Up regulated	3.37165	PH01000623G0280	Down regulated	-4.08341
PH01162702G0010	Up regulated	3.32269	PH01000507G0180	Down regulated	-6.75418
PH01007960G0010	Up regulated	3.53161	**Glucose-1-phosphate adenylyltransferase (glgC)**		
PH01001102G0130	Up regulated	1.79413	PH01000983G0230	Down regulated	-3.77544
**Trehalose 6-phosphate synthase (TPS)**			PH01003937G0060	Down regulated	-2.69411
PH01002176G0130	Up regulated	2.05552	PH01001244G0260	Down regulated	-1.96907
PH01002098G0280	Up regulated	1.08285	PH01000903G0520	Down regulated	-2.74907
PH01001492G0440	Up regulated	1.56593	PH01004879G0040	Down regulated	-4.85015
PH01000453G0440	Up regulated	1.04673	PH01001253G0400	Down regulated	-1.10561
PH01000798G0100	Up regulated	1.25648	**Starch synthase (glgA)**		
**Trehalose 6-phosphate phosphatase (TPP)**			PH01000804G0500	Down regulated	-2.08870
PH01002176G0130	Up regulated	2.05552	PH01000135G1340	Down regulated	-1.08300
PH01002098G0280	Up regulated	1.08285	PH01001914G0350	Down regulated	-1.76234
PH01001492G0440	Up regulated	1.56593	PH01000065G1160	Down regulated	-3.41950
PH01000453G0440	Up regulated	1.04673	PH01000814G0170	Down regulated	-1.22160
PH01000798G0100	Up regulated	1.25648	**Cellulase (CELB)**		
**Alpha, alpha-trehalase (TRE)**			PH01001459G0050	Down regulated	-1.85693
PH01000362G0480	Up regulated	1.60479	PH01000332G1080	Down regulated	-2.19068
**Glycogen synthase (GYS)**			PH01003005G0220	Down regulated	-4.49288
PH01001692G0230	Down regulated	-1.24309	PH01001590G0100	Down regulated	-2.56356
**Granule-bound starch synthase (WAXY)**			**Beta-glucosidase (bglB)**		
PH01000269G0880	Down regulated	-1.49334	PH01000513G0030	Down regulated	-5.08625
**1,4-alpha-glucan branching enzyme (GBE1)**			PH01002028G0390	Down regulated	-3.22951
PH01000019G0310	Down regulated	-3.94720	PH01002028G0330	Down regulated	-5.92525
PH01002662G0130	Down regulated	-1.71463	PH01005398G0010	Down regulated	-2.47312
PH01002470G0090	Down regulated	-4.47197	PH01000650G0660	Down regulated	-1.26806
PH01001371G0200	Down regulated	-1.18469	PH01000650G0640	Down regulated	-2.01122
**4-alpha-glucanotransferase (malQ)**					
PH01000936G0470	Down regulated	-3.98938			
PH01000068G1680	Down regulated	-1.78807			

Table S6. Summary of sequencing data of mitochondrial transcriptome and alignment information of clean reads.

Sample	Total clean reads	Mitochondrial genome of *Bambusa Oldhamii*	
		Total mapping ratio	Uniquely mapping ratio
M1_1	30,665,518	1.64%	1.62%
M1_2	37,327,478	2.55%	2.53%
M1_3	41,806,922	2.46%	2.43%
M2_1	29,416,610	2.00%	1.97%
M2_2	45,573,976	3.45%	3.44%
M2_3	44,839,246	3.41%	3.39%
Mean value	38,271,625	2.62%	2.56%

Table S7. Comparison of protein-coding gene content between *Phyllostachys edulis* mitochondrial transcriptome and other grasses mitochondrial genomes.

	Phyllostachys edulis **mitochondrial transcriptome**	**mitochondrial genomes**				
		Bambusa oldhamii	***Ferrocalamus rimosivaginus***	***Oryza sativ***	***Triticum aestivum***	**Zea mays**
**Complex I**						
nd1,2,3,4, 4L,5,6,7,9	+	+	+	+	+	+
**Complex II**						
sdh3, 4	–	–	–	–	–	–
**Complex III**						
cob	+	+	+	+	+	+
**Complex IV**						
cox1,2,3	+	+	+	+	+	+
**Complex V**						
atp1,4,6,8,9	+	+	+	+	+	+
**Cytochrome c biogenesis**						
ccmB,C,FC,FN	+	+	+	+	+	+
**Ribosomal**						
rpl2	Φ	Φ	Φ	+	–	–
rpl5	+	+	+	+	+	–
rpl16	+	+	+	+	+	+
rps1,2,3,4,7,12,13	+	+	+	+	+	+
rps14	Φ	Φ	Φ	Φ	Φ	–
rps19	+	+	+	+	Φ	–
**Other ORFs**						
matR	+	+	+	+	+	+
mttB	+	+	+	+	+	+

+, presence of the gene; –, absence of the gene; Φ, pseudogene.

Table S8. DEPs related to starch and sucrose metabolism in total proteome.

Gene ID	Level	Log FC	Gene ID	Level	Log FC
**Trehalose 6-phosphate synthase (TPS)**			**Glucose-1-phosphate adenylyltransferase (glgC)**		
PH01000007G2760	Down regulated	-2.563	PH01003937G0060	Down regulated	-1.962
PH01000670G0310	Down regulated	-2.085	PH01000983G0230	Down regulated	-2.158
**Granule-bound starch synthase (WAXY)**			**Starch synthase (glgA)**		
PH01000439G0530	Down regulated	-10.116	PH01000804G0500	Down regulated	-3.654
PH01000269G0880	Down regulated	-3.740	PH01001914G0350	Down regulated	-11.473
**Beta-glucosidase (bglB)**			PH01000065G1160	Down regulated	-5.878
PH01005398G0010	Up regulated	10.721	PH01001203G0570	Down regulated	-11.637
PH01002028G0390	Up regulated	2.768	**4-alpha-glucanotransferase (malQ)**		
PH01000513G0030	Up regulated	2.390	PH01000936G0470	Down regulated	-3.915
